# New flavonoid – *N*,*N*-dibenzyl(*N*-methyl)amine hybrids: Multi-target-directed agents for Alzheimer´s disease endowed with neurogenic properties

**DOI:** 10.1080/14756366.2019.1581184

**Published:** 2019-03-07

**Authors:** Martín Estrada-Valencia, Clara Herrera-Arozamena, Concepción Pérez, Dolores Viña, José A. Morales-García, Ana Pérez-Castillo, Eva Ramos, Alejandro Romero, Erik Laurini, Sabrina Pricl, María Isabel Rodríguez-Franco

**Affiliations:** a Institute of Medicinal Chemistry, Spanish Council for Scientific Research (IQM-CSIC), Madrid, Spain;; b Centre for Research in Molecular Medicine and Chronic Diseases (CIMUS), University of Santiago de Compostela, Santiago de Compostela, Spain;; c Institute for Biomedical Research "Alberto Sols", Spanish Council for Scientific Research (IIB-CSIC), Madrid, Spain;; d Biomedical Research Networking Centre on Neurodegenerative Diseases (CIBERNED), Madrid, Spain;; e Department of Cellular Biology, Medical School, Complutense University of Madrid, Madrid, Spain;; f Department of Pharmacology and Toxicology, Faculty of Veterinary Medicine, Complutense University of Madrid, Madrid, Spain; x; g Molecular Biology and Nanotechnology Laboratory (MolBNL@UniTS), Department of Engineering and Architecture (DEA), Trieste, Italy

**Keywords:** Multi-target-directed ligands, neurogenesis, sigma receptors, human β-secretase, human lipoxygenase-5, human cholinesterases, Alzheimer’s disease, neurodegenerative diseases

## Abstract

The design of multi-target directed ligands (MTDLs) is a valid approach for obtaining effective drugs for complex pathologies. MTDLs that combine neuro-repair properties and block the first steps of neurotoxic cascades could be the so long wanted remedies to treat neurodegenerative diseases (NDs). By linking two privileged scaffolds with well-known activities in ND-targets, the flavonoid and the *N*,*N*-dibenzyl(*N*-methyl)amine (DBMA) fragments, new CNS-permeable flavonoid – DBMA hybrids (**1**–**13**) were obtained. They were subjected to biological evaluation in a battery of targets involved in Alzheimer’s disease (AD) and other NDs, namely human cholinesterases (hAChE/hBuChE), β-secretase (hBACE-1), monoamine oxidases (hMAO-A/B), lipoxygenase-5 (hLOX-5) and sigma receptors (σ_1_R/σ_2_R). After a funnel-type screening, 6,7-dimethoxychromone – DBMA (**6**) was highlighted due to its neurogenic properties and an interesting MTD-profile in hAChE, hLOX-5, hBACE-1 and σ_1_R. Molecular dynamic simulations showed the most relevant drug-protein interactions of hybrid **6**, which could synergistically contribute to neuronal regeneration and block neurodegeneration.

## Introduction

Despite the great advances achieved in the understanding of the Alzheimer’s disease (AD) pathophysiology, our current knowledge about this illness is still an incomplete puzzle. Based on different causative factors, several hypotheses have tried to explain its origin, such as the amyloid-beta peptide (Aβ) accumulation, abnormal *tau* phosphorylation, cholinergic transmission deficits, exacerbated neuroinflammatory response and oxidative damage. Nevertheless, up to date, all of them are just loose pieces of the puzzle, and none of them is able to account for the complexity of AD^1^.

This brings us to the main fact that is clear today, the multifactorial nature of this disease, in which different factors contribute to its onset and progression. However, the current approved drugs are mainly active at a single target, acetylcholinesterase (AChE) or *N*-methyl-D-aspartate receptor (NMDA), that have barely been able to modify the disease progression^2^. This failure lies in the complex network of pathophysiological processes underlying the origin of the AD-related neurodegeneration, and in our lack of knowledge about the primordial event that triggers the others, if there is only one. So far, we understand that genetic, epigenetic and environmental factors are involved in neurodegeneration. Moreover, increasing evidence suggests that some systemic alterations in AD should be understood as the echo of underlying processes related to the origin of the disease and not only as secondary effects of neuronal death. Some of these systemic alterations include abnormalities in immunity and antioxidant responses, metabolic disorders, hepatic dysfunction, cardiovascular diseases and gut microbiota disturbance, among others^3^. This reveals that a multifactorial process such as AD cannot be stopped or prevented with a treatment based on a single and simple mechanism of action.

From these ideas, more holistic strategies must be explored, not only regarding to the pharmacological approach but to the whole body of research that is developing around the world related to neurodegeneration. From a pharmacological point of view, the multi-target directed ligand (MTDL) strategy has emerged as an alternative against the traditional single target – single molecule approach^4^^,^^5^. Indeed, in the last decade an increasing number of new drugs based on this paradigm have been developed for the treatment of several complex diseases. In the field of NDs, safinamide was approved in Europe in February 2015 and in the United States in March 2017 for the treatment of Parkinson's disease (PD), due to its MTD-profile that combines dopaminergic (MAO-B and dopamine reuptake inhibition) and non-dopaminergic properties (blockade of voltage-dependent Na^+^ and Ca^2+^ channels)^6^. Interestingly, to achieve maximum efficiency in stopping or delaying neurodegeneration, the MTD drugs must hit targets located upstream in the neurotoxic cascades^7^^,^^8^.

Different post-mortem studies in AD patients have shown a great increase in the peroxidation of brain biomolecules^9^, suggesting that oxidative damage is an early event that precedes the formation of abnormal protein aggregates and that antioxidant drugs could be useful for preventing such injuries^10^. On other hand, levels of monoamine oxidases (MAO-A and MAO-B) are increased in neurodegenerative pathologies, such as AD and PD^11^. The activities of these enzymes contribute to the neurodegenerative process by promoting the formation of the harmful Aβ peptide and by increasing the oxidative stress (OS) through the production of hydrogen peroxide^12^. Consequently, MAOs’ inhibitors could decrease both the generation of amyloid plaques and radical oxygen/nitrogen species (ROS/RNS)^13^.

Sequential cleavage of the amyloid precursor protein by β- and γ-secretases produces pathologic Aβ peptides, which are prone to aggregate into amyloid plaques^14^. The fact that β-secretase, also known as β-site amyloid-precursor-protein-cleaving enzyme 1 (BACE-1), is located upstream in the amyloid cascade makes its inhibitors interesting AD disease-modifying drugs^15^. In recent years, BACE-1 has gained great importance because several clinical trials have shown a correlation between the inhibition of this enzyme and low levels of pathological Aβ peptides^16^^,^^17^. In spite of the above, several potent BACE-1 inhibitors have failed in different clinical trials. For instance, verubecestat (MK-8931) a potent BACE-1 inhibitor, able to reduce Aβ levels in cerebrospinal fluid up to 81%, was ineffective in AD patients ranging from 55 to 85 years in phase III studies^18^. Although these negative results may lead us to reconsider the validity of the amyloid hypothesis, the results of current clinical trials of BACE-1 inhibitors in asymptomatic individuals at risk to develop AD or with prodromal AD are still to be seen^19^. From another point of view, issues associated to BACE-1 inhibitors could be related to the fact that they are administered to AD patients in the severe stages where they cannot provide any therapeutic benefit, or to the fact that these drugs are still based on the single-target paradigm. The situation is so complex that the ideal drug for AD should be administered in the right moment and has to be able to reduce neuronal death coming from almost all sources of toxicity: oxidative stress, misfolded proteins, excitotoxicity and so on. Therefore, we still consider important the search for new BACE-1 inhibitors, but endowed with a MTD-profile in light of the current literature^20^.

Lipoxygenase-5 (LOX-5) is an enzyme widely distributed in central nervous system (CNS), mainly in neurons and glia. Two especially vulnerable regions to neurodegeneration, the cerebral cortex and the hippocampus, possess the highest expression levels of this enzyme that is upregulated in AD patients^21^. LOX-5 plays a key role in inflammatory processes, which *per se* may be an important reason for its pharmacological modulation, and interestingly, overexpression of LOX-5 in the AD-triple transgenic mouse model (3xTg) leads to a clear exacerbation of memory deficits and increased burdens of both *tau* and amyloid deposits^22^. Conversely, 3xTg mice treated with the LOX-5 inhibitor zileuton present an improvement in memory, cognition, synaptic integrity and a reduction in amyloid and *tau* pathologies^23^. These findings establish a functional role of LOX-5 in the AD-pathogenesis, pointing out the interest of LOX-5 inhibitors as valuable therapeutic agents, as they reduce neuro-inflammation and the main AD-hallmarks, amyloid plaques and neurofibrillary tangles.

On the other hand, the sigma-1 receptor (σ_1_R) is a chaperone-like receptor located at the mitochondria-associated endoplasmic reticulum membrane, widely distributed in CNS and implicated in memory, emotional and cognitive processes. While the complete biological role of this receptor remains unknown, it has been discovered to regulate the function of a variety of processes through opioid, NMDA, dopaminergic and cholinergic receptors. Pharmacological or genetic invalidation of σ_1_R enhances Aβ toxicity^24^, whereas its activation exerts protection against OS by stimulation of the antioxidant response elements and subsequent transcription of the proteins involved in the cellular response to oxidative damage^25^.

Although the adult neurogenic processes are restricted to specific small brain regions and a large characterisation of their extent and relevance is still needed^26^^,^^27^, the pharmacological induction of neurogenesis is achievable and may significate a great opportunity to help the brain to recover its own self-renewal capacity^28^^,^^29^. Maybe the so desired disease-modifying AD-drug would include the ability to induce the differentiation of neural stem cells into mature neurons capable to replace those lost by neurodegeneration. In this regard, a promising compound is the steroid allopregnanolone that has demonstrated to promote neurogenic processes and reverse cognitive deficits in a mouse model of AD^30^ and that recently completed phase-I studies^31^.

In the last years, a part of our work has been focussed on the design of new compounds with a MTD-profile aiming at some of the most important pharmacological objectives related to AD and NDs. Apart from the classical targets (AChE, BACE-1, MAOs), other important proteins involved in NDs have been explored, such as σ_1_R, LOX-5 and the activation of neurogenic processes^32–37^. Continuing with our interest in MTDLs, in this work we describe the synthesis of new flavonoid-based hybrids (**1**–**13**) and their biological evaluation in a battery of ND-targets, namely hAChE, hBACE-1, hMAOs, hLOX-5 and σ_1_R, and in a phenotypic assay for assessing neurogenic properties.

**Figure 1.**
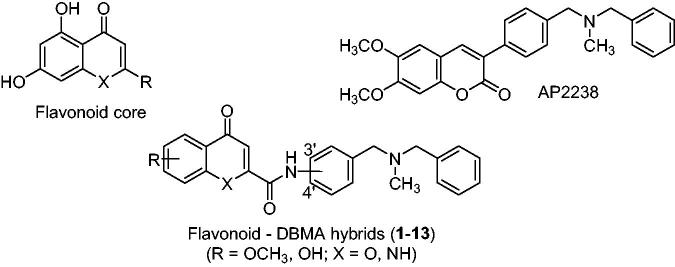
Structures of flavonoids, AP2238 and flavonoid – *N*,*N*-dibenzyl(*N*-methyl)amine hybrids (**1**–**13**).

New hybrids were designed by linking two privileged chemotypes with well-known therapeutic activities in AD and other NDs: (i) a flavonoid core derived from 4-chromenone or 4-quinolone, with potential neurogenic properties^38^ and inhibition of BACE-1^39^, LOX-5^40^ and MAO^41^; and (ii) the *N*,*N*-dibenzyl(*N*-methyl)amine (DBMA) fragment, present in AP2238^42–44^ and other AD-directed MTDLs^45^^,^^46^, due to its proved interaction with the catalytic anionic site (CAS) of AChE^47^ (Figure 1).

## Materials and methods

### Chemistry

Reagents and solvents were purchased from common commercial suppliers, mostly Sigma-Aldrich, and were used without further purification. Thin-layer chromatography (TLC) was carried out using Merck silica gel 60 F254 plates and compounds were visualised under UV-light (λ = 254 or 365 nm) and/or stained with phosphomolybdic acid 10% wt. in ethanol. Automatised chromatographic separation was carried out in an IsoleraOne (Biotage) equip, using different silica Biotage ZIP KP-Sil 50 μ cartridges. High-performance liquid chromatography was performed on a Waters analytical HPLC-MS (Alliance Watters 2690) equipped with a SunFire C_18_ 4.6 × 50 mm column, a UV photodiode array detector (λ = 214–274 nm) and quadrupole mass spectrometer (Micromass ZQ). HPLC analyses were used to confirm the purity of all compounds (≥ 95%) and were performed on Waters 6000 equipment, at a flow rate of 1.0 ml/min, with a UV photodiode array detector (λ = 214–274 nm), and using a Delta Pak C_18_ 5 µm, 300 Å column. The elution was performed in a gradient mixture of acetonitrile (ACN)/water, starting in most of cases with 15% and ending with 95% of ACN within 5 min (Water – ACN (1 5 →95%), g.t. 5 min). Melting points were determined in a MP70 apparatus (Mettler Toledo). ^1^H NMR and ^13 ^C NMR spectra were obtained in MeOD, DMSO-d_6_ CDCl_3_ or CD_3_OD solutions using Varian INOVA-300, Varian INOVA-400, Varian Mercury-400 or Varian Unity-500 spectrometers. High resolution mass spectrometry (HRMS) data were obtained by electron spray ionisation in positive mode (ESI^+^) using a Hewlett-Packard MSD 1100 spectrometer.

#### Synthesis of chromene – DBMA hybrids (general method A)

The corresponding 4-oxo-4*H*-chromene-2-carboxylic acid (1.0 mmol) and CDI (1.3 mmol) were mixed into a 10 ml mw vial under N_2_ atmosphere. The vial was sealed up and 5 ml of anhydrous DMF were added using a syringe to dissolve the mixture (CO_2_↑). This solution was heated into an mw reactor at 120 °C during 10 min to complete the activation of the acid. Afterward, a solution of the corresponding amine (1.2 mmol) in 2 ml of DMF was added with a syringe; this final solution was heated during 10 min at 150 °C to obtain the desired amide. After completion of the reaction, the DMF was evaporated under reduced pressure; the crude material was re-dissolved in 25 ml of EtOAc and washed with water (5 × 5 ml), brine (3 × 5 ml), dried over MgSO_4_ and concentrated. The product was purified by column chromatography using EtOAc:MeOH (9:1) as eluent.

#### Synthesis of phenolic derivatives (method B)

Under N_2_ atmosphere, to a solution of the corresponding methoxy hybrid (0.1 mmol) in anhydrous DCM (3 ml), BBr_3_ (1 equivalent per each heteroatom present in the molecule) was added slowly under magnetic stirring. The mixture was allowed to react overnight at RT and then, quenched with MeOH (dropwise until end of effervescence). Solvent was evaporated under reduced pressure and MeOH addition was repeated several times until no fumes were observed. The residue was purified by column chromatography using a gradient of EtOAc/MeOH 0 → 10% as eluent.

#### Synthesis of 4-oxo-1,4-dihydroquinoline – DBMA hybrids (general method C)

Under N_2_ atmosphere, to a mixture of the corresponding methyl 4-oxo-1,4-dihydroquinoline-2-carboxylate (1.0 mmol) and the corresponding amine (2.5 mmol) in dry THF (3.5 ml) in a mw vial, Al(CH_3_)_3_ (2 M in heptane, 3.0 mmol) was injected with a syringe. This mixture was heated into an mw reactor at 120 °C during 1.5 min and then, the crude material was treated with HCl 2 M (dropwise) until the end of gas generation, neutralised with NaOH 2 M and the liquid phase evaporated to dryness. The solid was washed with EtOAc (5 × 5.0 ml) and MeOH (2 × 5.0 ml) and these fractions were mixed and concentrated under reduced pressure. The product was purified by column chromatography using a gradient of EtOAc in hexane (0 → 65%) as eluent.

***N-(4-((Benzyl(methyl)amino)methyl)phenyl)-6-methoxy-4-oxo-4H-chromene-2-carboxamide (1)***. White solid, yield: 77% (method A); mp 146-147 °C. ^1^H NMR (300 MHz, CDCl_3_) δ 8.55 (s, 1H, NH), 7.67 (d, *J* = 8.5 Hz, 2H, H_2'_), 7.59 (d, *J* = 3.1 Hz, 1H, H_5_), 7.54 (d, *J* = 9.1 Hz, 1H, H_7_), 7.42 (d, *J* = 8.5 Hz, 2H, H_3'_), 7.39 – 7.29 (m, 5H, H_8,_*_o, m_*), 7.28 – 7.23 (m, 2H, H_3,_*_p_*), 3.91 (s, 3H, 6-OMe), 3.54 (s, 2H, H_β_), 3.53 (s, 2H, H_α_), 2.20 (s, 3H, H_γ_) (Figure S1). ^13 ^C NMR (75 MHz, CDCl_3_) δ 178.02 (C_4_), 157.78 (C_6_), 157.06 (C_9_), 154.52 (C_2_), 150.02 (C_8a_), 139.28 (C*_i_*), 137.14 (C_4'_), 135.26 (C_1'_), 129.86 (C_3'_), 129.05 (C*_m_*), 128.39 (C*_p_*), 127.13 (C*_o_*), 125.24 (C_4a_), 124.90 (C_8_), 120.47 (C_2'_), 119.60 (C_7_), 111.84 (C_3_), 105.34 (C_5_), 61.99 (C_β_), 61.37 (C_α_), 56.16 (C, 6-OMe), 42.37 (C_γ_) (Figure S2). HRMS [ESI+] *m/z* = 428.1754 [M]^+^, calculated for [C_26_H_24_N_2_O_4_]^+^ 428.1736 (Figure S3). HPLC purity 99%.

**Figure 2.**
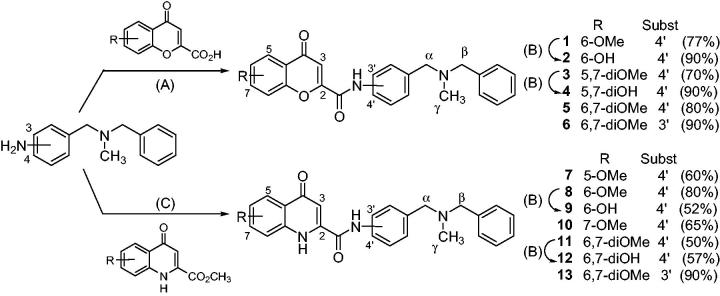
Synthesis of flavonoid – *N*,*N*-dibenzyl(*N*-methyl)amine hybrids (**1**–**13**). Reagents and conditions: (A) CDI, DMF, mw 120 °C, 10 min; (B) BBr_3_, DCM, r.t., overnight; (C) Al(CH_3_)_3_, THF, mw 120 °C, 3 min.

**Figure 3.**
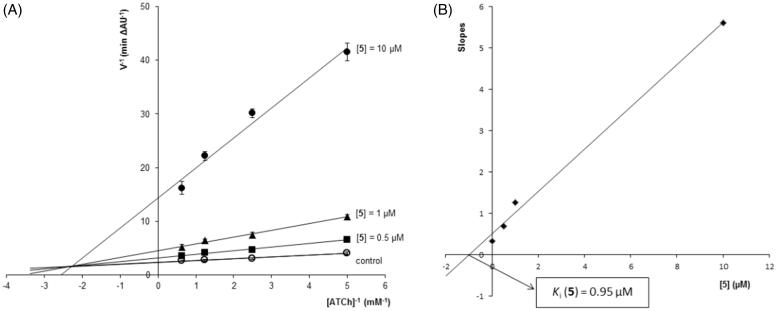
Kinetic study on the mechanism of hAChE inhibition by hybrid **5**. (A) Overlaid Lineweaver-Burk reciprocal plots of hAChE initial velocity at increasing substrate concentration (ATCh, 0.2–1.6 mM) in the absence of inhibitor and in the presence of **5** (0–10 µM) are shown. Lines were derived from a least-squares analysis of the data points. (B) Replot of slopes *vs.* inhibitor concentration for calculating *K*_i_ as the intersection in the x-axis.

***N-(4-((Benzyl(methyl)amino)methyl)phenyl)-6-hydroxy-4-oxo-4H-chromene-2-carboxamide (2)***. Yellow solid, yield: 90% (method B); mp 186-189 °C. ^1^H NMR (500 MHz, MeOD) δ 7.77 (d, *J* = 8.6 Hz, 2H, H_2'_), 7.73 (d, *J* = 9.1 Hz, 1H, H_8_), 7.45 (d, *J* = 2.9 Hz, 1H, H_5_), 7.41 (d, *J* = 8.6 Hz, 2H, H_3'_), 7.38 – 7.31 (m, 5H, H_7,_*_o, m_*), 7.29 – 7.25 (m, 1H, H*_p_*), 7.05 (s, 1H, H_3_), 3.56 (s, 4H, H_α, β_), 2.20 (s, 3H, H_γ_) (Figure S4). ^13 ^C NMR (126 MHz, MeOD) δ 180.41 (C_4_), 159.57 (C_9_), 157.38 (C_2_), 157.29 (C_8a_), 151.03 (C_6_), 139.51 (C*_i_*), 137.75 (C_1'_), 136.84 (C_4'_), 130.92 (C*_o_*), 130.42 (C_2'_), 129.36 (C*_m_*), 128.36 (C*_p_*), 126.09 (C_4a_), 125.57 (C_7_), 122.39 (C_3'_), 121.32 (C_8_), 111.16 (C_3_), 108.72 (C_5_), 62.65 (C_β_), 62.13 (C_α_), 42.30 (C_γ_) (Figure S5). HRMS [ESI+] *m/z* = 414.1583 [M]^+^, calculated for [C_25_H_22_N_2_O_4_]^+^ 414.1580 (Figure S6). HPLC purity 100%.

**Figure 4.**
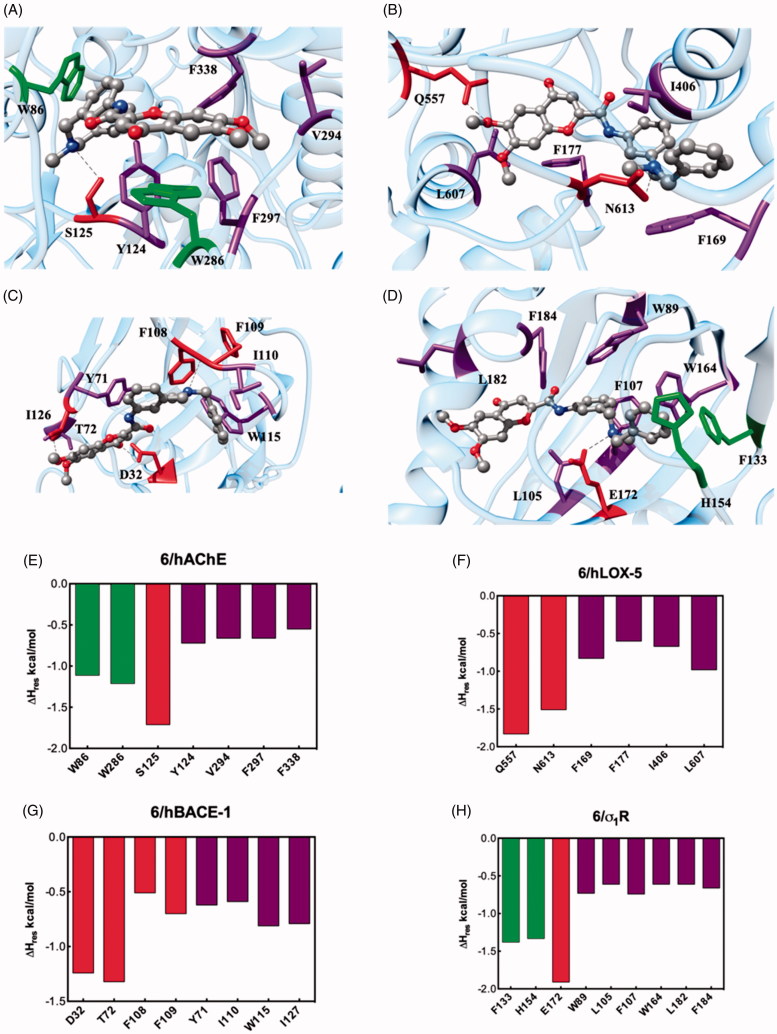
Upper panel: Details of compound **6** in the binding pocket of the hAChE (A), hLOX-5 (B), hBACE-1 (C), and σ_1_R (D). Compound **6** is shown as atom-coloured sticks-and-balls (C, grey, N, blue, O, red) while the side chains of proteins residues mainly interacting with **6** are depicted as coloured sticks and labelled. Hydrogen bonds are shown as black broken lines. Hydrogen atoms, water molecules, ions, and counterions are omitted for clarity. Lower panel: Per-residue binding free energy decomposition of the main involved amino acids of the complex between **6** with hAChE (E), hLOX-5 (F), hBACE-1 (G) and σ_1_R (H).

**Figure 5.**
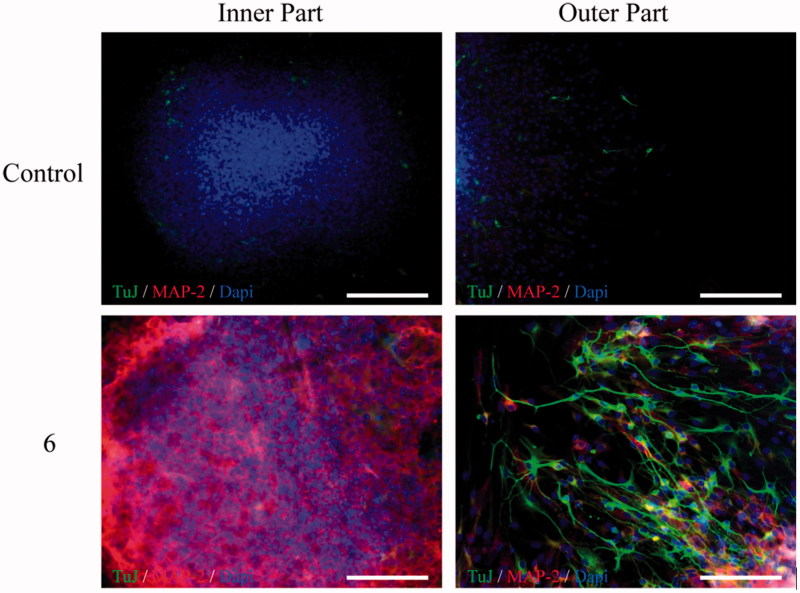
*In vitro* neurogenic effects of chromone-based hybrid **6** (10 µM) on adult mice SGZ-derived NSCs. In the presence of compound **6**, NS were grown for 7 days and allowed to differentiate for 3 additional days. Immunocytochemical analysis shows the expression of two neuronal markers: TuJ1 clone (green) and MAP-2 (red), in the inner and outer part of the NS. DAPI was used for nuclear staining. Scale bar: 20 µm.

***N-(4-((Benzyl(methyl)amino)methyl)phenyl)-5,7-dimethoxy-4-oxo-4H-chromene-2-carboxamide (3)***. White solid, yield: 70% (method A); mp 99-101 °C. ^1^H NMR (300 MHz, CDCl_3_) δ 8.81 (s, 1H, NH), 7.88 (d, *J* = 8.4 Hz, 2H, H_2'_), 7.60 (d, *J* = 8.4 Hz, 2H, H_3'_), 7.56 – 7.41 (m, 5H, Ph), 7.26 (s, 1H, H_3_), 6.75 (d, *J* = 2.3 Hz, 1H, H_6_), 6.58 (d, *J* = 2.3 Hz, 1H, H_8_), 4.11 (s, 3H, 7-OMe), 4.09 (s, 3H, 5-OMe), 3.73 (s, 2H, H_β_), 3.72 (s, 2H, H_α_), 2.39 (s, 3H, H_γ_) (Figure S7). ^13 ^C NMR (75 MHz, CDCl_3_) δ 176.96 (C_4_), 164.84 (C_7_), 161.28 (C_5_), 159.02 (C_8a_), 157.16 (C_9_), 152.57 (C_2_), 139.22 (C*_i_*), 136.85 (C_4'_), 135.48 (C_1'_), 129.81 (C_3'_), 129.05 (C*_o_*), 128.38 (C*_m_*), 127.13 (C*_p_*), 120.47 (C_2'_), 114.39 (C_3_), 109.85 (C_4a_), 96.86 (C_6_), 93.03 (C_8_), 61.94 (C_β_), 61.37 (C_α_), 56.55 (C, 5-OMe), 56.05 (C, 7-OMe), 42.34 (C_γ_) (Figure S8). HRMS [ESI+] *m/z* = 458.1863 [M]^+^, calculated for [C_27_H_26_N_2_O_5_]^+^ 458.1842 (Figure S9). HPLC purity 99%.

***N-(4-((Benzyl(methyl)amino)methyl)phenyl)-5,7-dihydroxy-4-oxo-4H-chromene-2-carboxamide (4)***. Bright yellow solid, yield: 90% (method B); mp 199-200 °C. ^1^H NMR (500 MHz, MeOD) δ 7.75 (d, *J* = 8.5 Hz, 2H, H_3'_), 7.41 (d, *J* = 8.5 Hz, 2H, H_2'_), 7.39 – 7.33 (m, 4H, H*_o_*, *_m_*), 7.30 – 7.26 (m, 1H, H*_p_*), 6.94 (s, 1H, H_3_), 6.63 (d, *J* = 2.1 Hz, 1H, H_8_), 6.28 (d, *J* = 2.1 Hz, 1H, H_6_), 3.59 (s, 4H, H_α, β_), 2.22 (s, 3H, H_γ_). ^13 ^C NMR (126 MHz, MeOD) δ 183.60 (C_4_), 167.44 (C_7_), 163.42 (C_5_), 159.15 (C_9_), 159.00 (C_8a_), 157.45 (C_2_), 139.22 (C*_i_*), 137.77 (C_4'_), 136.59 (C_1'_), 130.98 (C_2'_), 130.48 (C*_o_*), 129.40 (C*_m_*), 128.46 (C*_p_*), 122.50 (C_3'_), 111.22 (C_3_), 106.32 (C_4a_), 100.92 (C_6_), 95.94 (C_8_), 62.60 (C_α_), 62.07 (C_β_), 42.22 (C_γ_). HRMS [ESI+] *m/z* = 430.1517 [M]^+^, calculated for [C_25_H_22_N_2_O_5_]^+^ 430.1529. HPLC purity 100%.

***N-(4-((Benzyl(methyl)amino)methyl)phenyl)-6,7-dimethoxy-4-oxo-4H-chromene-2-carboxamide (5)***. White solid, yield: 80% (method A); mp 119-120 °C. ^1^H NMR (300 MHz, MeOD) δ 7.89 (d, *J* = 8.6 Hz, 2H, H_3'_), 7.53 – 7.35 (m, 9H, Ph, H_2', 5, 8_), 7.06 (s, 1H, H_3_), 4.04 (s, 3H, 6-OMe), 3.98 (bs, 4H, H_α, β_), 3.95 (s, 3H, 7-OMe), 2.49 (s, 3H, H_γ_). ^13 ^C NMR (75 MHz, MeOD) δ 179.39 (C_4_), 159.50 (C_9_), 157.48 (C_6_), 156.76 (C_2_), 153.49 (C_8a_), 150.17 (C_7_), 139.17 (C_4'_), 136.52 (C_1'_), 134.41 (C*_i_*), 131.99 (C_2'_), 131.33 (C*_o_*), 129.93 (C*_m_*), 129.84 (C*_p_*), 122.53 (C_3'_), 118.53 (C_4a_), 111.87 (C_3_), 104.71 (C_5_), 101.48 (C_8_), 61.77 (C_α_), 61.30 (C_β_), 57.14 (C, 6-OMe), 56.69 (C, 7-OMe), 40.87 (C_γ_). HRMS [ESI+] *m/z* = 458.1848 [M]^+^, calculated for [C_27_H_26_N_2_O_5_]^+^ 450.1842. HPLC purity 100%.

***N-(3-((Benzyl(methyl)amino)methyl)phenyl)-6,7-dimethoxy-4-oxo-4H-chromene-2-carboxamide (6)***. White solid, yield: 90% (method A); mp 105-108 °C. ^1^H NMR (400 MHz, MeOD) δ 7.79 (bs, 1H, H_2'_), 7.71 (dd, *J* = 8.2, 1.6 Hz, 1H, H_4'_), 7.46 (s, 1H, H_5_), 7.39 – 7.31 (m, 6H, H*_o, m, p,_*_8_), 7.29 – 7.21 (m, 1H, H_5'_), 7.20 (dt, *J* = 8.2, 1.5 Hz, 1H, H_6'_), 7.03 (s, 1H, H_3_), 4.01 (s, 3H, 6-OMe), 3.92 (s, 3H, 7-OMe), 3.55 (bs, 4H, H_α, β_), 2.19 (s, 3H, H_γ_) (Figure S10). ^13 ^C NMR (101 MHz, MeOD) δ 179.43 (C_4_), 159.40 (C_9_), 157.41 (C_6_), 156.94 (C_2_), 153.48 (C_8a_), 150.11 (C_7_), 140.93 (C*_i_*), 139.68 (C_1'_), 138.69 (C_3'_), 130.40 (C*_o_*), 129.89 (C*_p_*), 129.34 (C*_m_*), 128.29 (C_5'_), 127.37 (C_6’_), 123.17 (C_2'_), 121.27 (C_4'_), 118.50 (C_4a_), 111.78 (C_3_), 104.68 (C_5_), 101.45 (C_8_), 62.79 (C_α_), 62.57 (C_β_), 57.10 (C, 6-OMe), 56.67 (C,/-OMe), 42.42 (C_γ_) (Figure S11). HRMS [ESI+] *m/z* = 458.1840 [M]^+^, calculated for [C_27_H_26_N_2_O_5_]^+^ 458.1841 (Figure S12). HPLC purity 100%.

***N-(4-((Benzyl(methyl)amino)methyl)phenyl)-5-methoxy-4-oxo-1,4-dihydroquinoline-2-carboxamide (7)***. White solid, yield: 60% (method C); mp 263-265 °C. ^1^H NMR (500 MHz, CDCl_3_) δ 10.20 (s, 1H NH_10_), 9.88 (s, 1H, NH_1_), 7.80 (d, *J* = 8.3 Hz, 2H, H_2'_), 7.75 (s, 1H, H_3_), 7.73 (m, 1H, H_8_), 7.63 (t, *J* = 7.8 Hz, 1H, H_7_), 7.46 – 7.36 (m, 4H, H_3',_*_o_*), 7.34 (t, *J* = 7.5 Hz, 2H, H*_m_*), 7.29 – 7.26 (m, 1H, H*_p_*), 6.92 (dd, *J* = 7.8, 0.9 Hz, 1H, H_6_), 4.13 (s, 3H, 5-OMe), 3.57 (s, 4H, H_α, β_), 2.23 (s, 3H, H_γ_) (Figure S13). ^13 ^C NMR (126 MHz, CDCl_3_) δ 164.19 (C_4_), 162.09 (C_9_), 156.07 (C_5_), 152.06 (C_2_), 149.81 (C_8a_), 136.90 (C_4'_), 135.49 (C_1'_), 130.07 (C_7_), 129.88 (C_3'_), 129.23 (C*_o_*), 128.45 (C*_m_*), 127.25 (C*_p_*), 1213.37 (C_8_), 119.77 (C_2'_), 112.61 (C_4a_), 105.39 (C_6_), 104.34 (C_3_), 61.84 (C_α_), 61.49 (C_β_), 56.76 (C_51_), 42.24 (C_γ_) (C*i* not detected) (Figure S14). HRMS [ESI+] *m/z* = 427.1900 [M]^+^, calculated for [C_26_H_25_N_3_O_3_]^+^ 427.1896 (Figure S15). HPLC purity 99%.

***N-(4-((Benzyl(methyl)amino)methyl)phenyl)-6-methoxy-4-oxo-1,4-dihydroquinoline-2-carboxamide (8)***. White solid, yield: 60% (method C); mp 261-263 °C. ^1^H NMR (500 MHz, DMSO-d_6_) δ 10.61 (s, 1H, NH), 7.96 (d, *J* = 9.1 Hz, 1H_8_), 7.82 (d, *J* = 8.0 Hz, 2H, H_2'_), 7.49 (d, *J* = 2.9 Hz, 1H, H_5_), 7.40 (dd, *J* = 9.1, 2.9 Hz, 1H, H_7_), 7.38 – 7.30 (m, 7H, H_3, 3',_*_o, m_*), 7.28 – 7.23 (m, 1H, H*_p_*), 3.89 (s, 3H, 6-OMe), 3.50 (s, 2H, H_β_), 3.48 (s, 2H, H_α_), 2.08 (s, 3H, H_γ_) (Figure S16). ^13 ^C NMR (126 MHz, DMSO-d_6_) δ 157.07 (C_6_), 139.09 (C*_i_*), 136.99 (C_1'_), 134.98 (C_4'_), 128.98 (C_3'_), 128.60 (C*_m_*), 128.20 (C*_o_*), 126.88 (C*_p_*), 122.98 (C_7_), 120.18 (C_2'_), 60.97 (C_β_), 60.57 (C_α_), 55.43 (C, 6-OMe), 41.63 (C_γ_) (C_3_ and quaternary carbons of 4-oxo-1,4-dihydroquinoline ring not detected) (Figure S17). HRMS [ESI+] *m/z* = 427.1905 [M]^+^, calculated for [C_26_H_25_N_3_O_3_]^+^ 427.1896 (Figure S18). HPLC purity 99%.

***N-(4-((Benzyl(methyl)amino)methyl)phenyl)-6-hydroxy-4-oxo-1,4-dihydroquinoline-2-carboxamide (9)***. White solid, yield: 90% (method B); mp 248-251 °C. ^1^H NMR (300 MHz, DMSO-d_6_) δ 10.56 (s, 1H, NH), 7.90 (s, 1H), 7.82 (d, *J* = 8.3 Hz, 2H), 7.53 – 7.15 (m, 10H), 3.50 (s, 2H, CH_2_), 3.48 (s, 2H, CH_2_), 2.08 (s, 3H, CH_3_) (Figure S19). ^13 ^C NMR (75 MHz, DMSO-d_6_) δ 139.08 (C), 137.00 (C), 128.96 (CH), 128.58 (CH), 128.18 (CH), 126.86 (CH), 122.80 (CH), 120.14 (CH), 60.95 (CH_2_), 60.56 (CH_2_), 41.61(CH_3_) (Only one CH from the 4-chromenone system was detected in ^13 ^C NMR) (Figure S20). HRMS [ESI+] *m/z* = 413.1751 [M]^+^, calculated for [C_25_H_23_N_3_O_3_]^+^ 413.1739 (Figure S21). HPLC purity 99%.

***N-(4-((Benzyl(methyl)amino)methyl)phenyl)-7-methoxy-4-oxo-1,4-dihydroquinoline-2-carboxamide (10)***. White solid, yield: 62% (method C); mp 261-263 °C. ^1^H NMR (300 MHz, DMSO-d_6_) δ 11.73 (s, 1H, NH_1_), 10.63 (s, 1H, NH_10_), 8.01 (d, *J* = 9.0 Hz, 1H, H_5_), 7.79 (d, *J* = 8.1 Hz, 2H, H_3'_), 7.50 – 7.42 (m, 1H, H_8_), 7.40 – 7.29 (m, 6H, H_2',_*_o, m_*), 7.28 – 7.19 (m, 1H, H*_p_*), 6.99 (bs, 1H, H_6_), 6.86 (bs, 1H, H_3_), 3.87 (s, 3H, 7-OMe), 3.50 (s, 2H, H_β_), 3.49 (s, 2H, H_α_), 2.08 (s, 3H, H_γ_). ^13 ^C NMR (75 MHz, DMSO-d_6_) δ 177.03 (C_4_), 162.28 (C_7_), 160.65 (C_9_), 139.04 (C*_i_*), 136.88 (C_4'_), 135.22 (C_1'_), 128.98 (C_2'_), 128.59 (C*_m_*), 128.18 (C*_o_*), 126.87 (C*_p_*), 126.44 (C_5_), 120.43 (C_3'_), 113.96 (C_6_), 107.56 (C_3_), 100.31 (C_8_), 60.96 (C_β_), 60.53 (C_α_), 55.45 (C, 7-OMe), 41.59 (C_γ_). HRMS [ESI+] *m/z* = 427.1898 [M]^+^, calculated for [C_26_H_25_N_3_O_3_]^+^ 427.1896. HPLC purity 99%.

***N-(4-((benzyl(methyl)amino)methyl)phenyl)-6,7-dimethoxy-4-oxo-1,4-dihydroquinoline-2-carboxamide (11)***. White solid, yield: 35% (method C); mp 245-248 °C. ^1^H NMR (500 MHz, MeOD) δ 7.79 (d, *J* = 8.2 Hz, 2H, CH), 7.59 (s, 1H, CH), 7.42 (d, *J* = 8.2 Hz, 2H, CH), 7.48 – 7.25 (m, 7H,), 7.05 (bs, 1H, NH), 4.00 (s, 3H, Me), 3.96 (s, 3H, OMe), 3.70 (bs, 4H, 2xCH_2_), 2.30 (s, 3H, CH_3_). ^13 ^C NMR (126 MHz, MeOD) δ 156.16 (C), 150.33 (C), 138.73 (C), 137.98 (C), 134.68 (C), 131.33 (CH), 130.76 (CH), 129.57 (CH), 128.89 (CH), 122.06 (CH), 103.89 (CH), 62.32 (CH), 61.85 (CH), 56.70 (C, OMe), 56.48 (C, OMe), 41.82 (CH_3_) (Some CH’s and quaternary carbons were not detected). HRMS [ESI+] *m/z* = 457.2009 [M]^+^, calculated for [C_27_H_27_N_3_O_4_]^+^ 457.2002. HPLC purity 99%.

***N-(4-((benzyl(methyl)amino)methyl)phenyl)-6,7-dihydroxy-4-oxo-1,4-dihydroquinoline-2-carboxamide (12)***. White solid, yield: 90% (method B); mp 248-251 °C. ^1^H NMR (500 MHz, MeOD) δ 7.99 (d, *J* = 8.1 Hz, 2H, H_2'_), 7.64 (d, *J* = 8.1 Hz, 2H, H_3'_), 7.62 (s, 1H, H_3_), 7.60 – 7.55 (m, 2H, H*_m_*), 7.54 – 7.50 (m, 5H, H_5, 8,_*_o, p_*), 4.58 – 4.51 (m, 2H, H_α_), 4.39 – 4.30 (m, 2H, H_β_), 2.77 (s, 3H, H_γ_). ^13 ^C NMR (126 MHz, MeOD) δ 194.03 (C_4_), 170.55 (C_9_), 160.79 (C), 156.94 (C_7_), 150.15 (C_6_), 143.73 (C), 140.71 (C_1'_), 133.97 (C), 133.26 (C_3'_), 132.39 (C*_o_*), 131.31 (C*_p_*), 130.77 (C*_i_*), 130.45 (C*_m_*), 127.38 (C_4'_), 122.65 (C_2'_), 118.34 (C_8_), 111.41 (C), 105.80 (C_3_), 103.76 (C_5_), 60.76 (C_α_), 60.30 (C_β_), 39.55 (C_γ_). HRMS [ESI+] *m/z* = 429.1700 [M]^+^, calculated for [C_25_H_23_N_3_O_4_]^+^ 429.1689. HPLC purity 99%.

***N-(3-((Benzyl(methyl)amino)methyl)phenyl)-6,7-dimethoxy-4-oxo-1,4-dihydroquinoline-2-carboxamide (13)***. White solid, yield: 90% (method C); mp 131–133 °C. ^1^H NMR (500 MHz, MeOD) δ 7.92 (s, 1H, CH), 7.76 (dd, *J* = 7.6, 1.3 Hz, 1H, CH), 7.55 (s, 1H, CH), 7.50 – 7.38 (m, 7H, Ph, 2xCH,), 7.31 (s, 1H, CH), 7.27 (dt, *J* = 7.7, 1.3 Hz, 1H, CH), 4.04 – 4.00 (bs, 4H, 2xCH_2_), 3.99 – 3.98 (m, 3H, OMe), 3.94 (s, 3H, OMe), 2.51 (s, 3H, CH_3_) (Figure S22). ^13 ^C NMR (126 MHz, MeOD) δ 156.08 (C), 150.35 (C), 139.66 (CH), 131.43 (CH), 130.54 (CH), 129.98 (CH), 129.98 (CH), 127.84 (CH), 123.80 (CH), 122.42 (CH), 103.75 (CH), 61.75 (CH_2_), 61.58 (CH_2_), 56.68 (C, OMe), 56.45 (C, OMe), 41.00 (CH_2_) (Quaternary carbons not observed and just one CH from the 4-chromenone system detected in ^13 ^C NMR) (Figure S23). HRMS [ESI+] *m/z* = 457.2002 [M]^+^, calculated for [C_27_H_27_N_3_O_3_]^+^ 457.2001 (Figure S24). HPLC purity 99%.

### Biochemical studies

#### Inhibition of human acetyl- and butyrylcholinesterase (hAChE and hBuChE)

The Ellman method was followed, using human recombinant AChE and BuChE from human serum^48^. The assay solution consisted of 0.1 M phosphate buffer pH 8.0, 400 μM 5,5´-dithiobis(2-nitrobenzoic acid) (DTNB, Ellman’s reagent), 0.05 U/mL hAChE (Sigma Chemical Co.) or 0.024 U/mL hBuChE (Sigma Chemical Co.), and 800 μM acetylthiocholine iodide, or 500 μM butyrylthiocholine as the substrate of the enzymatic reaction, respectively. The compounds tested were added to the assay solution and preincubated with the enzyme for 5 min at 30 °C. After that period, the substrate was added. The absorbance changes at 412 nm were recorded for 5 min with a UV/Vis microplate spectrophotometer, Multiskan Spectrum, Thermo-Electron Co. The reaction rates were compared and the inhibition percentage due to the presence of test compound was calculated. The IC_50_ is defined as the concentration of each compound that reduces at 50% the enzymatic activity without any inhibitor.

#### Inhibition of human monoamine oxidases (hMAO-A and hMAO-B)

MAO inhibition measurements were evaluated following the general procedure previously described^49^. Briefly, test drugs and adequate amounts of recombinant hMAO-A or hMAO-B (Sigma-Aldrich, Spain) required and adjusted to oxidise 165 pmol of p-tyramine/min in the control group, were incubated for 15 min at 37 °C in a flat-black-bottom 96-well microtest plate (BD Biosciences, Franklin Lakes, NJ) placed in the dark fluorimeter chamber. The reaction was started by adding 200 mM Amplex Red reagent (Molecular Probes, Inc., Eugene, OR), 1 U/mL horseradish peroxidase, and 1 mM *p*-tyramine. Then, the production of resorufin was quantified at 37 °C in a multidetection microplate fluorescence reader (FLX800, Bio-Tek Instruments, Inc., Winooski, VT) based on the fluorescence generated (excitation, 545 nm; emission, 590 nm). The specific fluorescence emission was calculated after subtraction of the background activity, which was determined from wells containing all components except the hMAO isoforms, which were replaced by a sodium phosphate buffer solution.

#### Oxygen radical absorbance capacity assay (ORAC)

Antioxidant activities were measured using the ORAC method^50^, in a Polarstar Galaxy plate reader (BMG Labtechnologies GmbH, Offenburg, Germany) with 485-P excitation and 520-P emission filters. The equipment was controlled by the Fluorostar Galaxy software (version 4.11–0) for fluorescence measurement. 2,2'-Azobis-(amidinopropane) dihydrochloride (AAPH), (±)-6-hydroxy-2,5,7,8-tetramethylchromane-2-carboxylic acid (trolox) and fluorescein (FL) were purchased from Sigma-Aldrich. The reaction was carried out in 75 mM phosphate buffer (pH 7.4) and the final reaction mixture was 200 μL. Antioxidant (20 μL) and FL (120 μL; 70 mM, final concentration) solutions were placed in a black 96-well microplate (96 F untreat, Nunc). The mixture was pre-incubated for 15 min at 37 °C and then, AAPH solution (60 μL, 12 mM, final concentration) was added rapidly using a multichannel pipette. The microplate was immediately placed in the reader and the fluorescence recorded every minute for 80 min. The microplate was automatically shaken prior each reading. Samples were measured at eight different concentrations (0.1–1 μM). A blank (FL + AAPH in phosphate buffer) instead of the sample solution and eight calibration solutions using trolox (1–8 μM) were also carried out in each assay. All the reaction mixtures were prepared in duplicate, and at least three independent assays were performed for each sample. Raw data were exported from the Fluostar Galaxy Software to an Excel sheet for further calculations. Antioxidant curves (fluorescence *vs.* time) were first normalised to the curve of the blank corresponding to the same assay, and the area under the fluorescence decay curve (AUC) was calculated. The net AUC corresponding to a sample was calculated by subtracting the AUC corresponding to the blank. Regression equations between net AUC and antioxidant concentration were calculated for all the samples. ORAC values were expressed as trolox equivalents by using the standard curve calculated for each assay, where the ORAC value of trolox was taken as 1.0.

#### In vitro blood–brain barrier permeation assay (PAMPA-BBB)

Prediction of the brain penetration was evaluated using a parallel artificial membrane permeation assay (PAMPA-BBB), in a similar manner as previously described^36^^,^^46^^,^^51–53^. Pipetting was performed with a semi-automatic pipettor (CyBi®-SELMA) and UV reading with a microplate spectrophotometer (Multiskan Spectrum, Thermo Electron Co.). Commercial drugs, phosphate buffered saline solution at pH 7.4 (PBS), and dodecane were purchased from Sigma, Aldrich, Acros, and Fluka. Millex filter units (PVDF membrane, diameter 25 mm, pore size 0.45 μm) were acquired from Millipore. The porcine brain lipid (PBL) was obtained from Avanti Polar Lipids. The donor microplate was a 96-well filter plate (PVDF membrane, pore size 0.45 μm) and the acceptor microplate was an indented 96-well plate, both from Millipore. The acceptor 96-well microplate was filled with 200 μL of PBS: ethanol (70:30) and the filter surface of the donor microplate was impregnated with 5 μL of porcine brain lipid (PBL) in dodecane (20 mg mL^−1^). Compounds were dissolved in PBS: ethanol (70:30) at 100 μg mL^−1^, filtered through a Millex filter, and then added to the donor wells (200 μL). The donor filter plate was carefully put on the acceptor plate to form a sandwich, which was left undisturbed for 120 min at 25 °C. After incubation, the donor plate is carefully removed and the concentration of compounds in the acceptor wells was determined by UV-Vis spectroscopy. Every sample is analysed at five wavelengths, in four wells and at least in three independent runs, and the results are given as the mean ± standard deviation. In each experiment, 11 quality control standards of known BBB permeability were included to validate and normalise the analysis set.

#### Human BACE-1 inhibition assay

This experiment was carried out according to the protocol described by the manufacturer (Invitrogen) using a FRET assay^54^. Briefly, an APP-based peptide substrate (rhodamine-EVNLDAEFK-quencher, *K*m of 20 μM) carrying the Swedish mutation and containing a rhodamine as a fluorescence donor and a quencher acceptor at each end was used. The intact substrate is weakly fluorescent and becomes highly fluorescent upon enzymatic cleavage. The assays were conducted in 50 mM sodium acetate buffer, pH 4.5, in a final enzyme concentration (1 U/mL). The mixture was incubated for 60 min at 25 °C under dark conditions and then stopped with 2.5 M sodium acetate. Fluorescence was measured with a FLUOstar Optima (BMG Labtechnologies GmbH, Offenburg, Germany) microplate reader at 545 nm excitation and 585 nm emission.

#### Inhibition of human lipoxygenase-5 (hLOX-5)

The fluorescence-based enzyme method developed by Pufahl et al. was followed^55^, in 96-well microtiter plates. The assay solution consists of Tris buffer (50 mM, pH 7.5), ethylenediaminetetraacetic acid (EDTA, 2 mM), CaCl_2_ (2 mM), arachidonic acid (AA, 3 μM), ATP (10 μM), 2',7'-dichlorodihydrofluorescein diacetate (H_2_DCFDA, 10 μM), hLOX-5 (100 mU/well), bovine glutathione peroxidase (GPx, 25 mU/well) and reduced glutathione (GSH, 1 mM). Compounds to be tested were added to the test solution prior to AA and ATP, and pre-incubated for a period of 10 min at room temperature. Then, the AA and ATP substrates were added; the enzymatic reaction allowed to progress for 20 min and ended by the addition of 40 μL of acetonitrile. The fluorescence measurements (excitation: 485 nm; emission: 520 nm) were performed on a FLUOstar OPTIMA (BMG LABTECH, Offenburg, Germany). IC_50_ is defined as the concentration of compound that inhibits enzymatic activity by 50% over the control of untreated enzyme.

#### Binding assays at sigma-1 and sigma-2 receptors

For σ_1_R assay, the thawed membrane preparation of guinea pig brain cortex (about 100 µg of protein) were incubated for 120 min at 37 °C with 2 nM [^3^H]-(+)-pentazocine (PerkinElmer, specific activity 34.9 C_i_/mmol) in 50 mM Tris-HCl, pH 7.4, 0.5 ml final volume. Non-specific binding was defined in the presence of 10 µM of unlabelled (+)-pentazocine. The reaction was stopped by vacuum filtration through GF/B glass-fiber filters presoaked with 0.5% polyethylenimine, followed by rapid washing with 2 ml ice-cold buffer. Filters were placed in 3 ml scintillation cocktail and the radioactivity determined by liquid scintillation counting.

For σ_2_R assay, 150 µg of rat liver homogenate were incubated for 120 min at room temperature with 3 nM [^3^H]-DTG (PerkinElmer, specific activity 58.1 C_i_/mmol) in 50 mM Tris-HCl, pH 8.0, 0.5 ml final volume. (+)-Pentazocine (500 nM) was used to mask σ_1_R and to define non-specific binding, respectively.

Competition studies were done using at least 11 different concentrations of the ligand under investigation. As control, three increasing concentrations of unlabeled (+)-pentazocine (σ_1_R) or DTG (σ_2_R) were always included. The compounds were prepared as 10 mM stock solutions in 100% DMSO and diluted with Tris-HCl buffer on the day of the experiment. The final DMSO concentration in the incubation tubes was maintained at 0.1%^56^.

IC_50_ values and Hill’s coefficients *n_H_* were calculated by nonlinear regression using a four parameters curve-fitting algorithm of the GraphPad Prism software (v(0).6, La Jolla California USA), and are reported as the mean ± SEM of three separate determinations performed in duplicate. The corresponding *K_i_* values were obtained by means of the Cheng–Prusoff equation, using the *K_d_* values obtained in saturation experiments.

#### Molecular simulation details

All simulations were carried out using the Pmemd modules of Amber 18^57^, running on our own CPU/GPU calculation cluster. Molecular graphics images were produced using the UCSF Chimera package (v.1.10)^58^. All other graphs were obtained using GraphPad Prism (v. 6.0). The molecular structures of hAChE, of hLOX-5, and of hBACE-1 were obtained from the Protein Data Bank (pdb code: 4EY7^59^, pdb code: 3V99^60^, and pdb code: 1M4H^61^ respectively) while the optimised membrane-bound 3 D structure of the σ_1_ receptor was obtained starting from the available Protein Data Bank file (pdb code: 5HK1^62^) and following a procedure previously described^63^^,^^64^.

The optimised structures of **6** were docked into each protein identified binding pocket using Autodock 4.2.6/Autodock Tools 1.4.6^65^ on a win64 platform. The resulting docked conformations were clustered and visualised; then, the structure of each resulting complex characterised by the lowest Autodock interaction energy in the prevailing cluster was selected for further modeling. Each compound/protein complex obtained from the docking procedure was further refined in Amber 18 using the quenched molecular dynamics (QMD) method as previously described [see, for example^32^^,^^66–68^, and reference therein]. Next, the best energy configuration of each complex resulting from QMD was subsequently solvated by a cubic box of TIP3P water molecules^69^ extending at least 10 Å in each direction from the solute. The system was neutralised and the solution ionic strength was adjusted to the physiological value of 0.15 M by adding the proper amounts of Na^+^ and Cl^−^ ions. Each solvated system was relaxed (500 steps of steepest descent followed by 500 other conjugate-gradient minimisation steps) and then gradually heated to the target temperature of 298 K in intervals of 50 ps of constant volume-constant temperature (NVT) molecular dynamics (MD) simulations (Verlet integration method, time step 1.0 fs). The Langevin thermostat was used to control temperature. During this phase of MD, the protein was restrained with a force constant of 2.0 kcal/(mol Å), and all simulations were carried out with periodic boundary conditions. Subsequently, the density of the system was equilibrated via MD runs in the isothermal-isobaric (NPT) ensemble, with a time step of 1 fs. All restraints on the protein atoms were then removed, and each system was further equilibrated using NPT MD runs at 298vK. Three equilibration steps were performed (4 ns each, time step 2.0 fs). System stability was monitored by the fluctuations of the root-mean-square-deviation (RMSD) of the simulated position of the backbone atoms of the protein with respect to those of the initial protein model. The equilibration phase was followed by a data production run consisting of 50 ns of MD simulations in the NVT ensemble. Data collection was performed on over the last 20 ns of each equilibrated MD trajectory were considered for statistical data collections. 1000 trajectory snapshots were analysed for each **6**/receptor complex. The free energy of binding ΔG_bind_ between **6** and the target proteins was estimated by resorting to the well-validated Molecular Mechanics/Poisson–Boltzmann Surface Area (MM/PBSA) approach^70^ implemented in Amber 18. The per residue binding free energy decomposition (interaction spectra) was carried out using the Molecular Mechanics/Generalized Boltzmann Surface Area (MM/GBSA) approach^71^^,^^72^, and was based on the same snapshots used in the binding free energy calculation.

#### Study of theoretical medicinal chemistry alerts in free databases

SMILES code of hybrids **1**–**13** were uploaded in two databases, namely ZINC15 (http://zinc15.docking.org/)^73^ and SwissADME (http://www.swissadme.ch/)^74^. PAINs and aggregation results are gathered in Table S1 (Supplementary information).

#### Neurogenic assays

Adult (3 months old) male C57BL/6 mice were used following the animal experimental procedures previously approved by the Ethics Committee for Animal Experimentation of the CSIC in accordance with the European Communities Council, directive 2010/63/EEC and National regulations, normative 53/2013. Special care was taken to minimise animal suffering. Neural stem cells were isolated from the SGZ of the dentate gyrus of the hippocampus of adult mice and cultured as NS according to previously published protocols^75^^,^^76^. Neural stem cells grown as NS were treated for 7 days in culture with compound **6** (10 µM). Now, NS were adhered onto 100 μg/mL poly-L-lysine-coated coverslips and treated for 3 additional days in the presence of serum but in the absence of exogenous growth factors to induce differentiation^77^. Finally, the expression of neuronal markers was analysed by immunocytochemistry using antibodies linked to neurogenesis: β-III-tubulin polyclonal antibody (TuJ clone; Abcam), a protein expressed at early stages of neurogenesis and a monoclonal microtubule-associated protein type 2 (MAP-2) antibody, a classical marker of late neuronal maturation. To visualise primary antibodies Alexa-fluor-labeled secondary antibodies (Molecular probes) were used. Nuclei were stained with DAPI. Fluorescent representative images were acquired in a LSM710 laser scanning spectral confocal microscope (Zeiss). Confocal microscope settings were adjusted to produce the optimum signal-to-noise ratio.

#### In silico toxicity and metabolism predictions

To assess toxicity prediction, we use Derek Nexus v 6.0.1 (knowledge base 2018 1.1, species: human), which is a knowledge-based expert system by Lhasa Limited where toxicity predictions consider the presence of a toxicophore in the query structure, and are the result of two processes: evaluating alerts and estimating the likelihood of toxicity^78^. The likelihood levels in Derek Nexus in highest to lowest order are: certain, probable, plausible, equivocal, doubted, improbable, and impossible^79^.

To predict metabolism, we use Meteor Nexus v 3.1.0 (knowledge base 2018 1.0.0), a knowledge-based approach to rank metabolites based on known metabolic reactions^78^. To predict first metabolic step of the parent compound (hybrid **6**), we analysed the phase-I biotransformation pathways, combining two different methods^80^. A qualitative [absolute reasoning (AR)] and quantitative (site of metabolism (SOM) scoring) assessment was applied, selecting the matching metabolites. The AR evaluated the likelihood level for a biotransformation to occur, and the minimal likelihood level was settled in “plausible”, what means that the weight of evidence supports the proposition^79^. The SOM scoring method uses experimental data for compounds that match the same biotransformation, have similar molecular weights and are chemically similar around the site of metabolism to hybrid **6**.

#### Mutagenic and carcinogenic risk assessment

The International Conference on Harmonisation (ICH) of technical requirements for registration of pharmaceuticals for human use has developed a guideline for the assessment and control of mutagenic impurities to limit potential carcinogenic risk, ICH M7^81^. This guideline purposes to provide a framework to identify mutagenic alerts with computational toxicology assessment, it has to be performed using two complementary QSAR methodologies. To reach this objective, we have used an *in silico* prediction system from Lhasa ltd. (Leeds, UK), Derek Nexus v3.2.0 (expert rule-based methodology) and Sarah Nexus v3.0.0 (statistical-based methodology) to obtain a classification according to OECD Guidance Document on the Validation of (Q)SAR Models^82^.

## Results and discussion

### Chemistry

The common precursors for synthetising desired hybrids **1**–**13** were 3- or 4-((benzyl(methyl)amino)methyl)anilines (Figure 2), which were obtained in excellent yields following well-known procedures. The coupling reaction between the above-mentioned anilines and several substituted 4-oxo-4*H*-chromene-2-carboxylic acids, in a microwave oven (mw) at 120 °C, using 1,1'-carbonyldiimidazole (CDI) as activating agent, gave 4-chromenone hybrids **1**, **3**, **5**, and **6** in good yields (70–90%). The methoxy-bearing 4-quinolinone hybrids (**7**, **8**, **10**, **11**, and **13**) were obtained in 50–90% yield by the Al(CH_3_)_3_-mediated amide formation between the corresponding aniline precursor and several substituted methyl 4-oxo-1,4-dihydroquinoline-2-carboxylates, into a mw oven at 120 °C. Finally, hydroxyl substituted compounds (**2**, **4**, **9**, and **12**) were obtained by the overnight treatment of the corresponding methoxy derivative with BBr_3_ in THF at RT. For achieving good yields in these transformations (70–90%) it was necessary to use one BBr_3_ equivalent for each ether group to be cleavage plus an additional equivalent for each heteroatom present in the molecule, due to the well-known complexation ability of the boron atom^52^^,^^83^.

All 4-chromenone – and 4-quinolone – DBMA hybrids **1**–**13** were purified in silica gel cartridges using an automatic chromatographic equip (IsoleraOne, Biotage) and were characterised by their analytical (HPLC, HRMS) and spectroscopic data (^1^H NMR, ^13 ^C NMR). Complete NMR assignment of their hydrogen and carbon atoms were made by ^1^H – ^13 ^C two-dimensional diagrams, mainly HSQC (heteronuclear single quantum correlation) and HMBC (heteronuclear multiple bond correlation).

### Inhibition of human cholinesterases

Firstly, new hybrids **1**–**13** were tested as inhibitors of human cholinesterases, namely hAChE and hBuChE, following the Ellman method and using donepezil as reference drug^48^. As shown in Table 1, new hybrids are potent and selective inhibitors of hAChE, with IC_50_ values comprised between the one-digit-micromolar and the sub-micromolar range. In all cases, the inhibition of hBuChE was worse, with IC_50_ exceeding 10 µM. Chromone derivatives displayed better hAChE inhibition potencies than their 4-quinolone counterparts (e.g. compare the pairs **1***vs.***8**; **2***vs.***9**; **5***vs.***11**; **6***vs.***13**). The nature of the substituents in the 4-chromenone ring exerted only modest effects on the hAChE inhibition; from the most effective hybrid **5** (IC_50_=0.99 µM) derived from 6,7-dimethoxy-4-oxo-4*H*-chromene, the inhibitory potencies given by the substituents ranked as follows: 6-hydroxy ≥ 6-methoxy ≥ 5,7-dihydroxy > 5,7-dimethoxy.

**Table 1.** Inhibition of human acetylcholinesterase (hAChE) and human monoamine oxidases (hMAO-A and hMAO-B). Assessment of the oxygen radical absorbance capacity (ORAC, trolox equivalents) and the CNS-permeation (PAMPA-BBB assay).

Compd.	X	R	Substitution	hAChE^a^	hMAO-A^a^	hMAO-B^a^	ORAC^b^	PAMPA-BBB^c^
**1**	O	6-OMe	4'	1.5 ± 0.2	1.6 ± 0.4	59.8 ± 3.7	n.d.	17.1 ± 1.5
**2**	O	6-OH	4'	1.3 ± 0.3	7.0 ± 0.8	9.7 ± 1.6	1.6 ± 0.2	6.8 ± 0.4
**3**	O	5,7-diOMe	4'	3.2 ± 0.4	>100	>100	n.d.	19.4 ± 1.7
**4**	O	5,7-diOH	4'	1.9 ± 0.6	22.8 ± 1.5	>100	1.2 ± 0.1	14.7 ± 1.2
**5**	O	6,7-diOMe	4'	1.0 ± 0.2	9.0 ± 0.1	8.1 ± 0.4	n.d.	19.1 ± 1.5
**6**	O	6,7-diOMe	3'	4.5 ± 0.8	>100	>100	n.d.	18.8 ± 1.3
**7**	NH	5-OMe	4'	4.0 ± 0.4	>100	15.2 ± 1.0	n.d.	23.0 ± 2.1
**8**	NH	6-OMe	4'	2.3 ± 0.1	>100	>100	n.d.	24.8 ± 2.0
**9**	NH	6-OH	4'	3.1 ± 0.3	>100	>100	1.2 ± 0.1	8.4 ± 0.7
**10**	NH	7-OMe	4'	2.3 ± 0.2	>100	>100	n.d.	21.7 ± 1.9
**11**	NH	6,7-diOMe	4'	4.5 ± 0.3	>100	>100	n.d.	19.1 ± 1.5
**12**	NH	6,7-diOH	4'	>10	>100	>100	0.5 ± 0.1	8.8 ± 0.7
**13**	NH	6,7-diOMe	3'	>10	>100	>100	n.d.	15.1 ± 1.2
Donepezil				0.01 ± 0.002	n.d.	n.d.	n.d.	n.d.
(*R*)-Deprenyl				n.d.	68.7 ± 4.2	0.017 ± 0.002	n.d.	n.d.
Iproniazid				n.d.	6.7 ± 0.8	7.5 ± 0.4	n.d.	n.d.
Moclobemide				n.d.	161.4 ± 19.4	>100	n.d.	n.d.
Trolox				n.d.	n.d.	n.d.	1.0	n.d.

Results are expressed as the mean ± SEM (*n* = 3). ^a^IC_50_ (µM). ^b^Trolox equivalents (mmol of trolox/mmol of tested compound). ^c^Permeability in the CNS (*P*_e_, 10^−6 ^cm s^−1^).

### Inhibition of human monoamine oxidases and antioxidant properties

New 4-chromenone – DBMA and 4-quinolone – DBMA hybrids (**1**–**13**) were evaluated as inhibitors of human recombinant MAO’s, expressed in baculovirus containing cDNA inserts for hMAO-A and hMAO-B. The production of oxygen peroxide from a common substrate for both isoenzymes (*p*-tyramine) was quantified using the Amplex Red MAO assay kit^84^ and results are gathered in Table 1. Five derivatives inhibited hMAO’s in the micromolar range, whereas the rest of tested compounds were found to be inactive at 100 µM (the highest concentration tested). All active hybrids, displayed higher potency towards hMAO-A than several drugs currently in clinical use for treating AD, PD and depressive disorders, such as selegiline (IC_50_=68.7 µM) and moclobemide (IC_50_=161.4 µM)^85^.

The 4-chromenone derivatives **1** (R = 6-OMe) and **4** (R = 5,7-diOH) showed a preference of at least 4.4-times for hMAO-A compared to hMAO-B, whereas the 5-methoxy-4-quinolone hybrid **7** was 6.6-fold more active in hMAO-B than in hMAO-A. Otherwise, the 4-chromenone derivatives **2** (R = 6-OH) and **5** (R = 6,7-diOCH_3_) exhibited a balanced inhibitory activity towards hMAO-A and hMAO-B, with IC_50_s in the low-micromolar range, between 7.0 and 9.7 µM. Interestingly, these values are very close to the IC_50_s displayed by the well-known antidepressant iproniazid^86^ (Table 1).

Furthermore, the antioxidant activities of new hybrids were measured using the oxygen radical absorbance capacity assay (ORAC). Trolox, the aromatic part of vitamin E responsible for its scavenging properties, was used as internal standard with the arbitrary value of ORAC = 1.0. Results are expressed as trolox equivalents (mmol of trolox/mmol of tested compound) in a comparative scale that indicates if a compound is a better (ORAC > 1.0) or a worse scavenger (ORAC < 1.0) than vitamin E. In this assay, only hybrids bearing hydroxyl groups were tested, because we reasonably expected that methoxy-derivatives would not exhibit substantial radical capture capacities according to our previous experience in flavonoid derivatives^32^^,^^52^. Thus, all assayed hydroxyl hybrids displayed interesting ORAC values, ranging from 0.5 to 1.6-fold the trolox value. Best results were found in 6-hydroxy derivatives (**2** and **9**, ORAC = 1.6 and 1.2, respectively) and in the 5,7-dihydroxy-4-chromenone hybrid **4** (ORAC = 1.2). The ORAC value clearly dropped for the 4-quinolinone – based hybrid **12** with two adjacent hydroxyl groups in positions 6 and 7 (ORAC = 0.5).

### Kinetic analysis of hAChE inhibition

The most potent hAChE inhibitor **5** (IC_50_=0.99 µM) was selected for studying inhibition kinetics using the Lineweaver-Burk method. The initial velocity of enzymatic inhibition was measured at four concentrations of the substrate acetylthiocholine (ATCh, 0.2–1.6 mM), in absence and presence of increasing concentrations of inhibitor **5** (0.5–10 µM). For each inhibitor concentration, plotting reciprocals of velocity *vs.* ATCh concentration (1/V *vs.* 1/[ATCh]) gave straight lines that were fitted by least-squares analysis (Figure 3). As inhibitor concentration increased, both 1/*V_max_* (*y* intercept) and −1/*K_m_* (*x* intercept) also increased, meaning a mixture of competitive and non-competitive mechanisms in the enzymatic inhibition. This mixed pattern could be due to the simultaneous interaction of hybrid **5** with both CAS and peripheral anionic site (PAS) of hAChE. Replot of slopes *vs.* inhibitor concentration gave a straight line that was also fitted by least-squares analysis and whose intersection on the negative *x*-axis provided an estimated inhibition constant (*K_i_*) of 0.95 µM.

### Prediction of the CNS-permeation

To check if new compounds could be able to reach their CNS-targets, we used the *in vitro* parallel artificial membrane permeability assay for the blood-brain barrier (PAMPA-BBB) described by Di et al.^87^, and partially modified by us for testing molecules with limited water-solubility^36^^,^^46^^,^^51–53^. The passive CNS-permeation of new compounds **1**–**13** through a lipid extract of porcine brain was measured at room temperature. In each experiment, 11 commercial drugs of known brain permeability were also tested and their permeability values normalised to the reported PAMPA-BBB data. According to the patterns previously established in the bibliography^87^, compounds with *P*_e_ exceeding 4 × 10^−6 ^cm s^−1^ would be able to cross the blood-brain barrier (cns+), whereas those displaying *P*_e_ less than 2 × 10^−6 ^cm s^−1^ would not reach the CNS (cns-). All new 4-chromenone – and 4-quinolone – DBMA hybrids **1**–**13** showed permeability values above 4 × 10^−6 ^cm s^−1^ in this *in vitro* BBB model (Table 1) and thus, it is expected they could enter into the CNS for interacting with their biological targets.

### Inhibition of human BACE-1

New compounds were evaluated as inhibitors of the human recombinant BACE-1 protein, using the fluorescence resonance energy transfer (FRET) assay^88^. Firstly, all compounds were tested at 10 µM giving inhibition percentages below 35%, with the exception of the 6,7-dimethoxy-4-chromenone hybrid with a *meta*-substitution in the central benzene ring **6**, which blocked around the 80% of the enzymatic activity. Then, the IC_50_ of **6** was calculated from the plot of hBACE-1 activity *vs.* inhibitor concentrations (0.1–100 µM) giving a value of 6.7 ± 0.8 μM.

### Inhibition of human lipoxygenase-5 (hLOX-5)

A selection of new hybrids covering different structural motifs was assayed as inhibitors of hLOX-5, followed the method described by Pufahl et al.^55^. Two well-known inhibitors, namely (*R*,*S*)-zileuton and nordihydroguaiaretic acid (NDGA), were used as internal references and results are gathered in Table 2. Tested compounds were modest hLOX-5 inhibitors, the majority of them with IC_50_ values in the two-digit micromolar range.

**Table 2.** Inhibition of human hLOX-5 (IC_50_, µM)^a^.

Compd.	X	R	Substitution	IC_50_ (µM)
**1**	O	6-OMe	4'	12.4 ± 0.5
**2**	O	6-OH	4'	>100 (38%)
**5**	O	6,7-diOMe	4'	72.4 ± 2.2
**6**	O	6,7-diOMe	3'	30.4 ± 1.6
**8**	NH	6-OMe	4'	>100
**10**	NH	7-OMe	4'	82.8 ± 4.1
**13**	NH	6,7-diOMe	3'	36.6 ± 3.1
(*R*,*S*)-Zileuton				0.15 ± 0.03
NDGA				0.097 ± 0.019

a Results are the mean ± SEM from three independent experiments.

The best hLOX-5 inhibitor was the 6-methoxy-4-oxochromene hybrid **1** displaying an IC_50_ of 12.4 µM. Interestingly, minimal structural modifications, namely replacement of the methoxy by a hydroxyl group (**2**) or the change of the 4-oxo-chromene by a 4-oxoquinoline ring (**8**), led to inactive compounds.

### Studies on sigma receptors

A selection of the most active hybrids in the previous experiments, covering also different structural features, was assayed on sigma receptors using competition experiments with radioligands^56^. Mammalian σ_1_ and σ_2_ receptors were obtained from guinea pig brain and rat liver, respectively. (+)-Pentazocine (a σ_1_-selective ligand) and 1,3-di-*o*-tolylguanidine (DTG, a σ_2_-selective ligand) were also evaluated for comparative purposes. Independently from the position of substituents on the 4-oxo-chromene ring and from the relative *meta*- or *para*-substitution of central benzene, all tested hybrids showed selectivity for the σ_1_ subtype with *K*_i_s in the sub-micromolar range, whereas *K*_i_s for the σ_2_R displayed one-digit micromolar values (Table 3).

**Table 3.** Affinity and selectivity towards σ_1_ and σ_2_ receptors.

Compd.	X	R	Substitution	*K*_i_ (µM)^a^		Selectivity *vs.* σ_1_R^b^
				σ_1_R	σ_2_R	
**1**	O	6-OMe	4'	0.48 ± 0.05	1.78 ± 0.43	3.7
**2**	O	6-OH	4'	0.38 ± 0.05	1.60 ± 0.28	4.2
**4**	O	5,7-diOH	4'	0.51 ± 0.07	> 3.0	> 5.9
**6**	O	6,7-diOMe	3'	0.53 ± 0.07	1.30 ± 0.35	2.5
Pentazocine				0.015 ± 0.003	n.d.	
DTG				n.d.	0.054 ± 0.008	

^a^Results are expressed as *K*_i_ (µM) and are the mean ± SEM of the experiments repeated in triplicates. ^b^Selectivity *vs.* σ_1_R was calculated as *K*_i_σ_2_R/*K*_i_σ_1_R. DTG: 1,3-di-o-tolylguanidine.

### Study of the theoretical medicinal chemistry alerts

In order to choose the best candidate for neurogenic studies, we studied the medicinal chemistry alerts of new flavonoid – DBMA hybrids **1**–**13** in two free databases, namely ZINC15 (http://zinc15.docking.org/)^73^ and SwissADME (http://www.swissadme.ch/)^74^. According to the ZINC15 web site, none of the hybrids was highlighted as pan assay interference compound (PAINS) or aggregator (see Table S1 in Supplementary Information). However, in the SwissADME platform hybrid **12** was marked with a structural alert (catechol), according to the Brenk method^89^.

### Molecular modelling rationale for the binding of compound 6 against its target proteins

Since 6,7-dimethoxychromone – DBMA **6** resulted to be the new derivative with the most interesting MTD-profile, we carried out molecular dynamics (MD) simulations in order to describe the different binding mechanisms against its biological targets. Accordingly, a putative binding site for **6** was identified on hAChE, hLOX-5, hBACE-1 and σ_1 _R (Figure 4(A–D)), by applying a well-validated docking protocol^66–68^. Accordingly, MD simulations of the resulting **6**/protein complexes were carried out, and the corresponding ligand/protein free energy of binding (ΔG_bind_) were calculated via the MM/PBSA (Molecular Mechanics/Poisson-Boltzmann Surface Area) approach^70^, yielding values in agreement with relevant experimental activity or affinity.

Afterwards, through a per-residue binding free energy deconvolution (PRBFED) of the enthalpic terms (ΔH_res_), we were able to define and describe the different binding mechanisms of **6** to its target proteins (Figure 4(E–H)). The quantification of the single contribution of the main protein residues involved in ligand binding allowed us to rationalise the specific interaction within the different receptor cavities.

Starting from the esterase enzyme, the binding mode of **6** (Figure 4(A)) is fostered by a stable hydrogen bond between the *N*-methyl nitrogen atom with the hydroxyl group of the hAChE S125 side chain (ΔH_res_= −1.71 kcal/mol, Figure 4(E)). Furthermore, the *N*-benzyl ring is involved in a π-π interaction with W286 (ΔH_res_= −1.21 kcal/mol), while the flavonoid core of **6** stacks through the chromene moiety against the side chain of W86 (ΔH_res_= −1.11 kcal/mol). Finally, the **6**/hAChE complex is further stabilised in the putative binding site through hydrophobic interactions between the ligand and the side chains of Y124, V294, F297, and F338 (∑ΔH_res_= −2.59 kcal/mol, Figure 4(E)). The sum of these stabilising energies results in favorable ΔG_bind_ values of −7.63 ± 0.23 kcal/mol.

The binding free energy calculated for the **6**/hLOX-5 complex was slightly less favourable than that for hAChE, with a ΔG_bind_= −6.21 ± 0.25 kcal/mol. The docking pose of **6** (Figure 4(B)) within the hLOX-5 binding site reveals two stable hydrogen bonds between the DBMA derivative and the lipoxygenase: the basic nitrogen atom acts as acceptor and finds its donor counterpart in the amidic -NH of the side chain of N613 (ΔH_res_= −1.50 kcal/mol), while a methoxy group of **6** performs a hydrogen bridge with Q557 (ΔH_res_= −1.83 kcal/mol). Moreover, the aromatic and hydrophobic portions of **6** are nestled in a cavity surrounded by the hLOX-5 residues F169, F177, I406, and L607 (∑ΔH_res_= −3.08 kcal/mol, Figure 4(F)).

Molecular modelling procedure was further expanded to analyse the interaction between **6** and human hBACE-1. The favourable binding process thermodynamics reflects into a negative ΔG_bind_ of −7.21 ± 0.28 kcal/mol, supporting the good inhibitory activity of **6** against hBACE-1. In details, the chromene moiety of **6** is involved in two hydrogen bonds with the secretase enzyme (Figure 4(C)): the methoxy substituent interacts with the hydroxyl group of T72 (ΔH_res_= −1.32 kcal/mol, Figure 4(G)), whilst the acceptor carbonylic group engages its donor counterpart of D32 (ΔH_res_= −1.24 kcal/mol). A further hydrogen bond is detected between the N-CH_3_ group of **6** and the backbone amide oxygen atom between F108 and F109 (∑ΔH_res_= −1.21 kcal/mol). Additionally, unspecific and favourable contacts are been detected between the lipophilic scaffold of **6** and the secretase residues Y71, I110, W115, and I127 leaning the enzyme binding cavity (∑ΔH_res_= −2.81 kcal/mol).

We concluded our computational analysis with the description of the binding mode of **6** onto σ_1 _R (Figure 4(D)). The interaction spectrum resulting from the analysis of the corresponding MD trajectory (Figure 4(H)) reveals a prototypical pattern of ligand-based intermolecular interactions in the σ_1 _R cavity: (i) a π-π interaction between the *N*-benzyl ring with the aromatic side chain of F133 (ΔH_res_= −1.38 kcal/mol) and H154 (ΔH_res_= −1.33 kcal/mol); (ii) a permanent salt bridge between the basic *N*-methyl nitrogen atom of **6** and the COO^−^ group of E172 (ΔH_res_= −1.91 kcal/mol), and (iii) a favourable network of hydrophobic interactions provided by a good insertion of the DBMA derivatives into the σ_1 _R lipophilic cavity surrounded by the side chains of residues W89, L105, F107, W164, L182, and F184 (∑ΔH_res_= −3.95 kcal/mol, Figure 4(H)). This efficient intermolecular interaction scheme is reflected in the good affinity of **6** toward σ_1 _R, as testified by the favourable value of the free energy of binding ΔG_bind_= −8.08 ± 0.23 kcal/mol.

### Neurogenic studies

Compound **6** with an interesting MTD-profile in hAChE, hLOX-5, hBACE-1 and σ_1_R [IC_50_ (hAChE)=4.5 µM; IC_50_ (hLOX-5)=30 µM; IC_50_ (hBACE-1)=6.7 µM; and IC_50_ (σ_1_R)=0.53 µM], and without any medicinal chemistry alert, was selected for neurogenic experiments. Thus, we studied the capacity of the chromone-based hybrid **6** to promote neurogenic effects in a primary culture of neural stem-cells (NSCs), isolated from the subgranular zone (SGZ) of adult rats and grown as free-floating neurospheres (NS). Compound was added to NS cultures for 7 days and then, NS were fixed to a substrate and allowed to differentiate in the presence of **6** for a 3-days additional period. Then, we evaluated the expression of β-III-tubulin (TuJ-1 clone; green) and microtubule-associated protein 2 (MAP-2, red) antibodies to visualise early and late neuronal maturation, respectively. As shown in Figure 5, control (basal) experiments (vehicle-treated cultures) only showed a few positive cells for TuJ-1 or MAP-2, whereas in cultures treated with compound **6** the number of both TuJ-1 and MAP-2 marked cells was clearly increased. These results indicate that chromone-based hybrid **6** is able to induce the differentiation of NSCs to a neuronal phenotype *in vitro*.

### In silico toxicity and metabolism of hybrid 6

With the aim of advancing one step further in the study of the potential therapeutic success of hybrid **6**, we performed an *in silico* prediction of its toxicity and metabolism, using the Derek Nexus system^78^. The toxicity predictions obtained with Derek Nexus are based on the comparison of the structural features of a given compound with one or more toxicophore patterns (structural alerts) in human species using the Lhasa's knowledge database. Among the 57 toxicity endpoints analysed for hybrid **6**, for 56 of them no alerts were predicted at the minimum reasoning level of “impossible” (See Chart S1 in the Supplementary Information). Only *in vitro* inhibition of the human ether-a-go-go-related gene (hERG) potassium channel was considered “plausible”, although with a low-confidence under 67% according to Judson et al.^79^

To add value to the toxicological assessment of hybrid **6**, we performed an *in silico* prediction of its phase-I metabolism in humans, obtaining 10 matching metabolites (Table 4). Potential toxicity was also evaluated for these metabolites with Derek Nexus, in which the minimum likelihood to consider a toxic result in the analysis was "plausible". Results showed that six metabolites were not associated with any structural alerts for toxicity by Derek, and four (M3, M9, M10 and M19) were associated to a “plausible” hERG channel inhibition *in vitro*, also with a confidence under 67%.

**Table 4.**
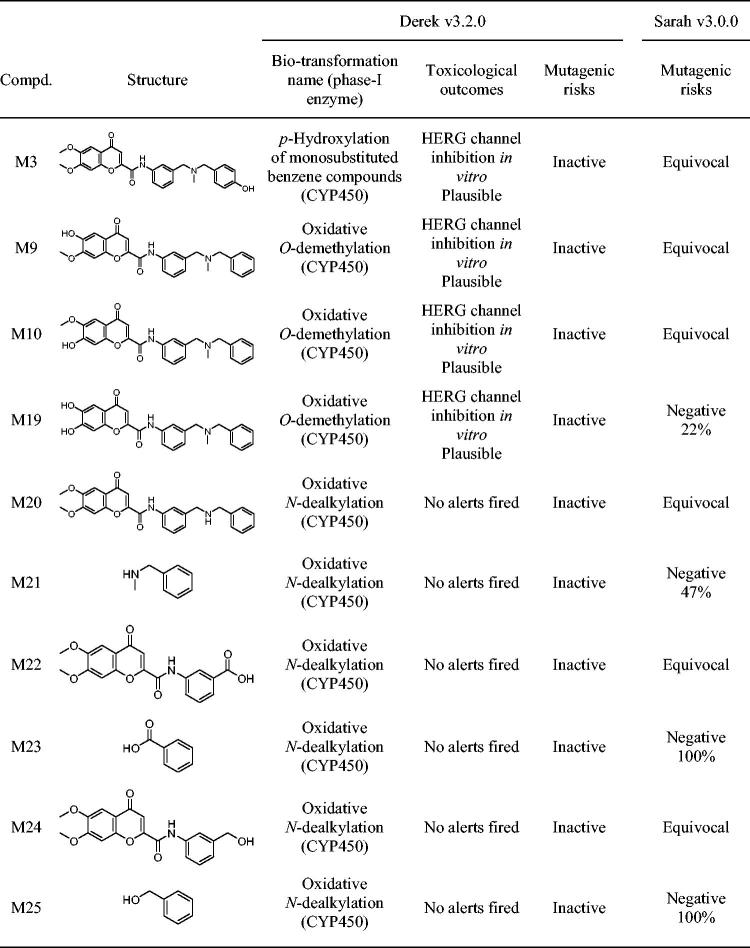
The high scored metabolites of hybrid **6** with reasoning levels of “plausible” and “probable” predictions. Biotransformation names, phase-I enzymes and toxicological effects predictions by Derek v3.2.0. Mutagenic risks assessment by two complementary QSAR methodologies (Derek v3.2.0 and Sarah v3.0.0).

Furthermore, each predicted metabolite was selected to evaluate its toxicity and mutagenic potential under the ICH M7 guideline^81^ and results are also gathered in Table 4. The carcinogenic risk assessment was carried out with two complementary QSAR methodologies, Derek (KB 2018 1.1) and Sarah (Model 2.0), which predicted an absence of structural alerts for four of the ten metabolites of the hybrid **6**. According to the ICH M7 guide, these results are sufficient to conclude that there is no mutagenic concern and no further tests are recommended for them (Class 5). However, six metabolites obtained an uncertain result, since Derek Nexus predicted a confident negative prediction, while Sarah gave an equivocal prediction. Nevertheless, the equivocal predictions by Sarah are not enough strong to overturn the negative result obtained by Derek. Whereas they are all based on negative results, they have low percentages of confidence, and the confidence level was settled below the 8% as equivocal.

For the parent compound hybrid **6**, in spite of hERG channel inhibition *in vitro*, no other toxicity alerts were fired. Four of the predicted metabolites also showed the same alert. This alert describes a structure-based pharmacophore developed primarily from compounds that have been reported to be inhibitors of the hERG potassium channel^90–92^. The blockage of this channel can lead to the lengthening of the ventricular repolarization phase in the heart, and is characterised on the electrocardiogram as a prolongation of the QT interval^93^. As this is a common feature that makes molecules fall in the preclinical phases, a more deeper investigation should be performed for hybrid **6** and its four metabolites prior to pass to advanced pharmacological assays.

## Conclusions

Thirteen new 4-chromenone – DBMA and 4-quinolone – DBMA hybrids were obtained by connecting flavonoid-related structures and an AP2238 fragment. In general, these MTDLs displayed selective inhibition of hAChE (IC_50_s = 0.99–4.5 µM) compared with hBuChE (IC_50_s > 10 µM) and were CNS-permeable according to the *in vitro* PAMPA-BBB assay. Some hybrids showed micromolar inhibition of hMAO-A (IC_50_s = 1.6–22.8 µM) and hMAO-B (IC_50_s = 8.1–59.8 µM), in many cases with IC_50_ values very close or better than the well-known antidepressants selegiline, iproniazid and moclobemide, concomitantly used in the treatment of AD and PD. Compounds bearing any hydroxyl group in the flavonoid core are good radical scavengers, in some cases 1.6- and 1.2-fold better than vitamin E. Flavonoid – DBMA hybrids were not able to inhibit hBACE-1, with the exception of compound **6** that showed an IC_50_ value of 6.7 µM. Several hybrids were determined to be micromolar inhibitors of hLOX-5, 4-chromenone derivatives being better than their 4-quinolone counterparts. Regarding sigma receptors, all tested hybrids showed affinity values in the micromolar and sub-micromolar scales, with a selectivity of at least 2.5-times in favour to the σ_1_R subtype (*K*_i_=0.4–0.5 µM) compared to the σ_2_R.

*N*-(3-((Benzyl(methyl)amino)methyl)phenyl)-6,7-dimethoxy-4-oxo-4*H*-chromene-2-carboxamide (**6**), with an interesting MTD-profile in hAChE, hLOX-5, hBACE-1 and σ_1_R [IC_50_ (hAChE)=4.5 µM; IC_50_ (hLOX-5)=30 µM; IC_50_ (hBACE-1)=6.7 µM; and IC_50_ (σ_1_R) = 0.5 µM], was selected for a phenotypic assay to study its capacity to promote neurogenic effects. In a primary culture of neural stem-cells from the SGZ of adult rats, hybrid **6** stimulated the differentiation of neural stem-cells to a neuronal phenotype and thus, this hybrid could be a therapeutic agent promoting brain auto-repair processes and blocking early steps of neurodegenerative cascades.

Molecular dynamics simulations of hybrid **6** in hAChE, hLOX-5, hBACE-1 and σ_1_R have shown the main interactions with these proteins, providing a rational about the experimental values obtained.

The toxicological alerts for hybrid **6** and its predicted metabolites were promising and relative safe profiles were expected. Nevertheless, the hERG channel inhibition *in vitro* alert was showed, although with a low-confidence below 67%. In next works, *in vivo* studies of hybrid **6** will be carried out to verify its therapeutic actions, its potential cardiac effects, as well as its toxicological behaviour.

## Supplementary Material

Supplemental Material

## Supplementary information

Supplementary information includes ^1^H-NMR, ^13^C-NMR and HRMS data of flavonoid – DBMA hybrids, and *in silico* predictions of their toxicity and metabolism.
