# Supplementary material for: New flavonoid – N,N-dibenzyl(N-methyl)amine hybrids: Multi-target-directed agents for Alzheimer´s disease endowed with neurogenic properties
Source: J Enzyme Inhib Med Chem. 2019 Mar 7;34(1):712–27. doi: 10.1080/14756366.2019.1581184 (PMC6407579; doi:10.1080/14756366.2019.1581184)
Supplement: Supplemental Material [file IENZ_A_1581184_SM0321.pdf]

Supplementary information for

New Flavonoid – *N*,*N*-Dibenzyl(*N*-methyl)amine Hybrids: Multi-Target-Directed Agents for Alzheimer´s Disease Endowed with Neurogenic Properties

Martín Estrada-Valencia, Clara Herrera-Arozamena, Concepción Pérez, Dolores Viña, José A. Morales-García, Ana Pérez-Castillo, Eva Ramos, Alejandro Romero, Erik Laurini, Sabrina Pricl, and María Isabel Rodríguez-Franco*

*Corresponding Author:
María Isabel Rodríguez-Franco, PhD
Instituto de Química Médica, Consejo Superior de Investigaciones Científicas (IQM-CSIC),
C/ Juan de la Cierva 3, 28006-Madrid, Spain
E-mail address: isabelrguez@iqm.csic.es
ORCID: http://orcid.org/0000-0002-6500-792X

List of Contents

Medicinal chemistry alerts of hybrids **1-13** S2

In silico study of toxicity of hybrid **6** S3

^{1}H-NMR, ^{13}C-NMR and HRMS data of selected flavonoid – DBMA hybrids S4

References S16

Medicinal chemistry alerts of hybrids 1-13

Table S1. Medicinal chemistry alerts of hybrids **1-13** according to ZINC15 (http://zinc15.docking.org/) [1] and SwissADME (http://www.swissadme.ch/) [2] web sites.

	Structure	SMILES	Aggregator[a]	PAINS Alerts[a,b]	Brenk Alerts[b]
**1**	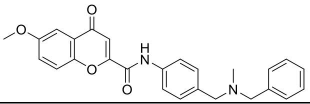	O=C(C1=CC(C2=C(O1)C=CC(OC)=C2)=O)NC3=CC=C(CN(CC4=CC=CC=C4)C)C=C3	No	0	0
**2**	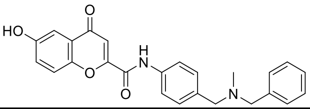	O=C(C1=CC(C2=C(O1)C=CC(O)=C2)=O)NC3=CC=C(CN(CC4=CC=CC=C4)C)C=C3	No	0	0
**3**	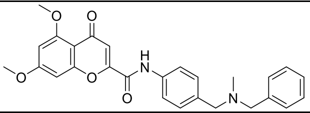	O=C(C1=CC(C2=C(O1)C=C(OC)C=C2OC)=O)NC3=CC=C(CN(CC4=CC=CC=C4)C)C=C3	No	0	0
**4**	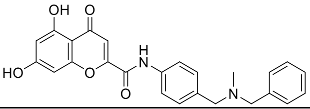	O=C(C1=CC(C2=C(O1)C=C(O)C=C2O)=O)NC3=CC=C(CN(CC4=CC=CC=C4)C)C=C3	No	0	0
**5**	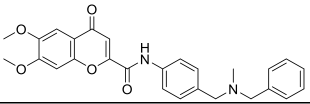	O=C(C1=CC(C2=C(O1)C=C(OC)C(OC)=C2)=O)NC3=CC=C(CN(CC4=CC=CC=C4)C)C=C3	No	0	0
**6**	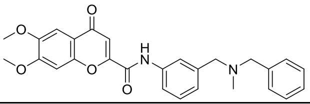	O=C(C1=CC(C2=C(O1)C=C(OC)C(OC)=C2)=O)NC3=CC=CC(CN(CC4=CC=CC=C4)C)=C3	No	0	0
**7**	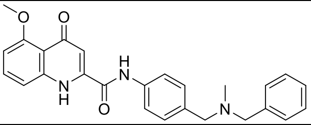	O=C(C(NC1=C2C(OC)=CC=C1)=CC2=O)NC3=CC=C(CN(CC4=CC=CC=C4)C)C=C3	No	0	0
**8**	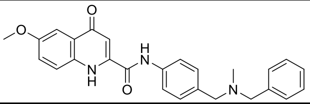	O=C(C(NC1=C2C=C(OC)C=C1)=CC2=O)NC3=CC=C(CN(CC4=CC=CC=C4)C)C=C3	No	0	0
**9**	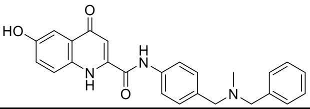	O=C(C(NC1=C2C=C(O)C=C1)=CC2=O)NC3=CC=C(CN(CC4=CC=CC=C4)C)C=C3	No	0	0
**10**	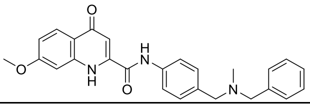	O=C(C(NC1=C2C=CC(OC)=C1)=CC2=O)NC3=CC=C(CN(CC4=CC=CC=C4)C)C=C3	No	0	0
**11**	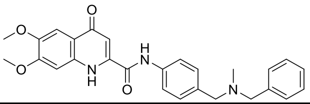	O=C(C(NC1=C2C=C(OC)C(OC)=C1)=CC2=O)NC3=CC=C(CN(CC4=CC=CC=C4)C)C=C3	No	0	0
**12**	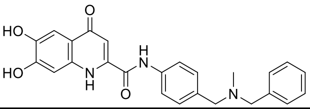	O=C(C(NC1=C2C=C(O)C(O)=C1)=CC2=O)NC3=CC=C(CN(CC4=CC=CC=C4)C)C=C3	No	0	1 (catechol)
**13**	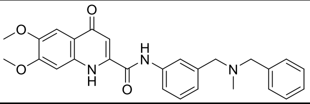	O=C(C(NC1=C2C=C(OC)C(OC)=C1)=CC2=O)NC3=CC=CC(CN(CC4=CC=CC=C4)C)=C3	No	0	0

[a] ZINC15 (http://zinc15.docking.org/). [b] SwissADME (http://www.swissadme.ch/).

In silico study of toxicity of hybrid 6

Chart S1. Toxicity endpoints predicted at the level of "impossible" for hybrid **6** after *in silico* prediction with Derek Nexus v6.0.1 [3].

5-αReductase inhibition	Mitochondrial dysfunction
Adrenal gland toxicity	Mutagenicity *in vitro*
Alpha-2-mu-Globulin nephropathy	Mutagenicity *in vivo*
Anaphylaxis	Nephrotoxicity
Androgen receptor modulation	Neurotoxicity
Bladder disorders	Non-specific genotoxicity *in vitro*
Bladder urothelial hyperplasia	Non-specific genotoxicity *in vivo*
Blood in urine	Occupational asthma
Bone marrow toxicity	Ocular toxicity
Bradycardia	Oestrogen receptor modulation
Carcinogenicity	Oestrogenicity
Cardiotoxicity	Peroxisome proliferation
Cerebral oedema	Phospholipidosis
Chloracne	Photo-induced chromosome damage *in vitro*
Chromosome damage *in vitro*	Photo-induced non-specific genotoxicity *in vitro*
Chromosome damage *in vivo*	Photo-induced non-specific genotoxicity *in vivo*
Cumulative effect on white cell count and immunology	Photoallergenicity
Cyanide-type effects	Photocarcinogenicity
Developmental toxicity	Photomutagenicity *in vitro*
Glucocorticoid receptor agonism	Phototoxicity
Hepatotoxicity	Pulmonary toxicity
High acute toxicity	Respiratory sensitisation
Irreversible cholinesterase inhibition	Splenotoxicity
Irritation (of the gastrointestinal tract)	Teratogenicity
Kidney disorders	Testicular toxicity
Kidney function-related toxicity	Thyroid toxicity
Lachrymation	Uncoupler of oxidative phosphorylation
Methaemoglobinaemia	Urolithiasis

^{1}H-NMR, ^{13}C-NMR and HRMS data of selected flavonoid – DBMA hybrids

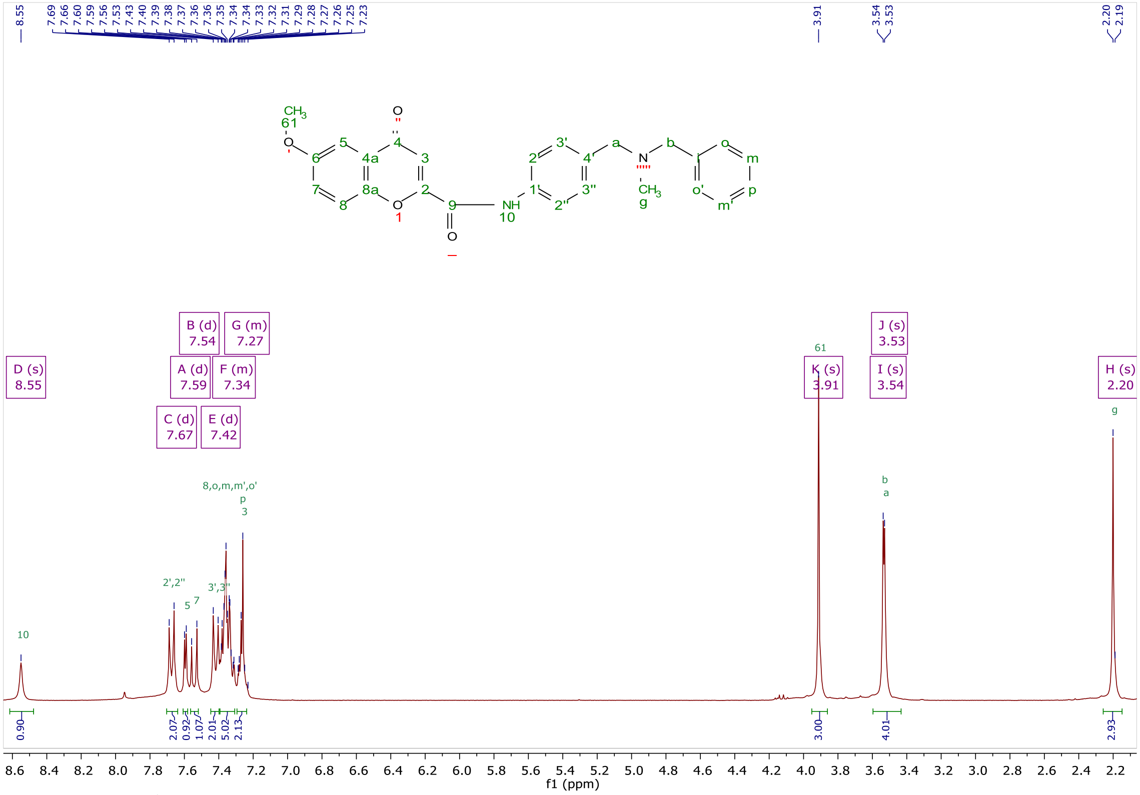

Figure S1. ^{1}H NMR of **1** in $CDCl_3$

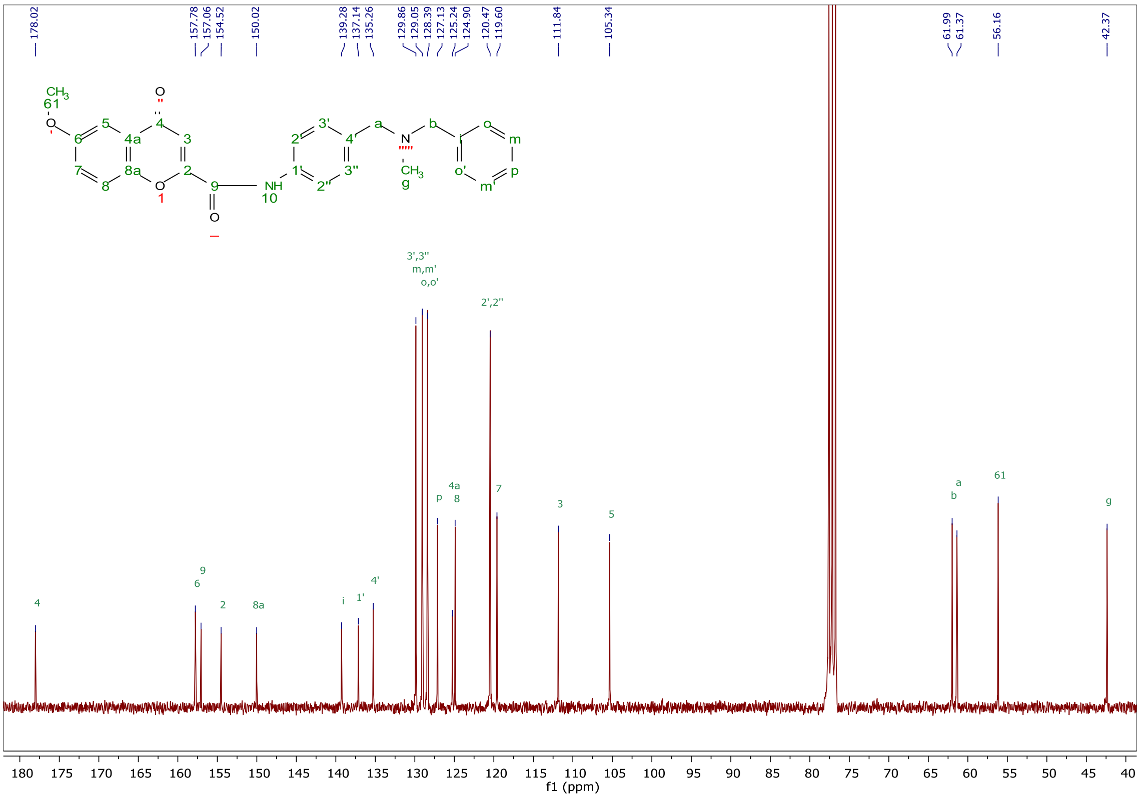

Figure S2. ^{13}C NMR of **1** in $CDCl_3$

**Data File**	5521_mev_303_01.d	**Sample Name**	mev_303
**Sample Type**	Sample	**Position**	Vial 22
**Instrument Name**	Instrument 1	**User Name**	
**Acq Method**	ESI_ACN_75_pos.m	**IRM Calibration Status**	Success
**DA Method**	Default.m	**Comment**	

Compound Table

Compound Label	RT	Mass	Abund	Formula	Tgt Mass	Diff (ppm)
Cpd 1: C26 H24 N2 O4	1.166	428.1754	29865	C26 H24 N2 O4	428.1736	4.17

Compound Label	RT	Algorithm	Mass
Cpd 1: C26 H24 N2 O4	1.166	Find By Formula	428.1754

MS Zoomed Spectrum

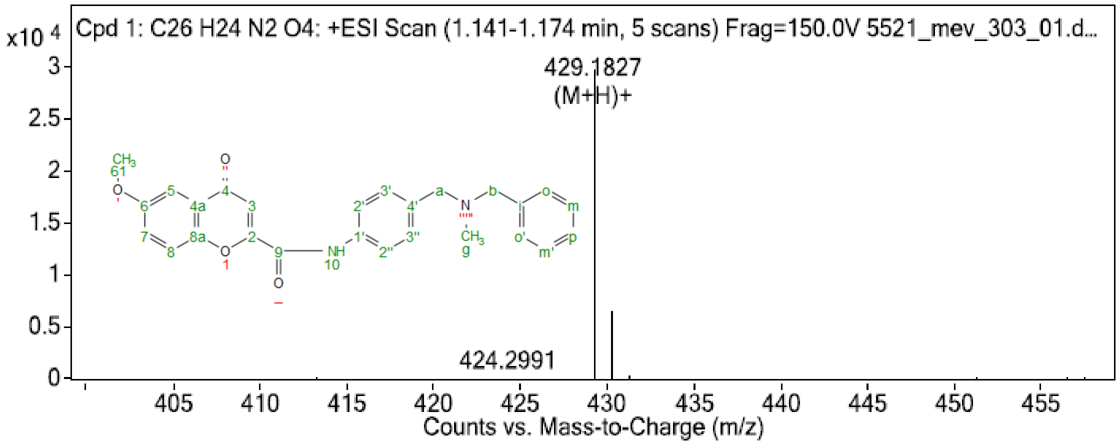

Figure S3. HRMS for hybrid **1**

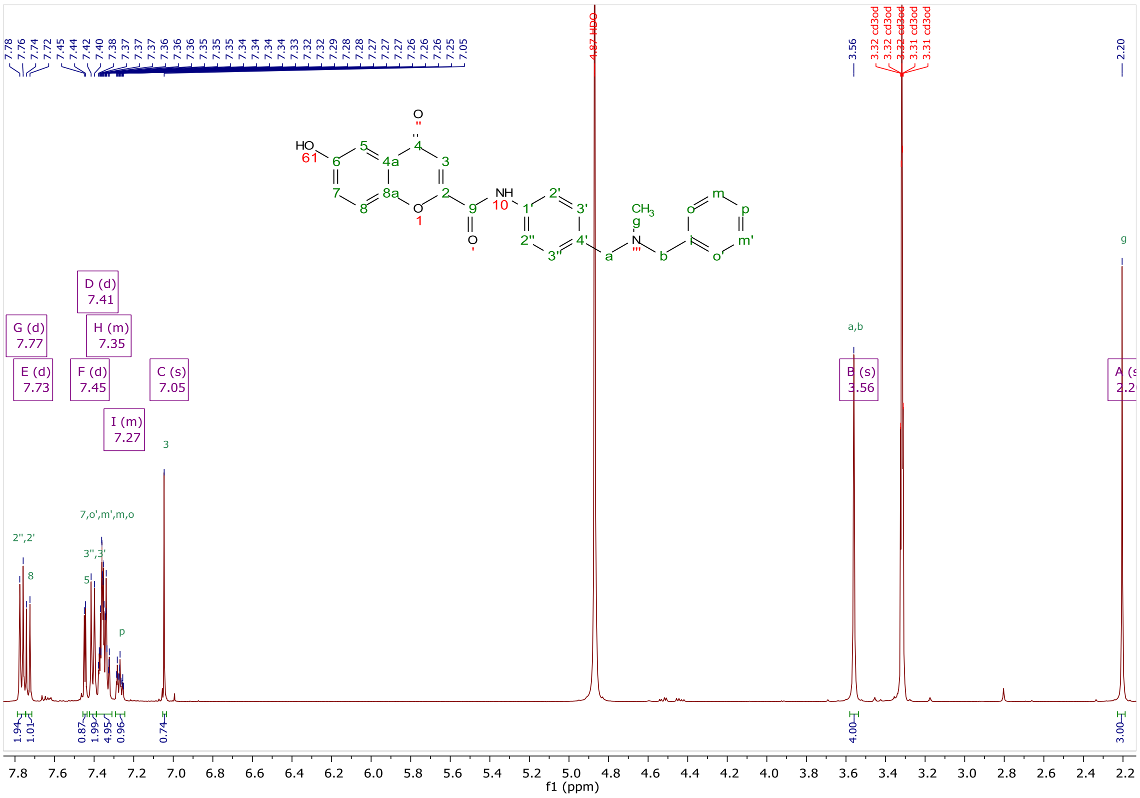

Figure S4. ^{1}H NMR of **2** in MeOD

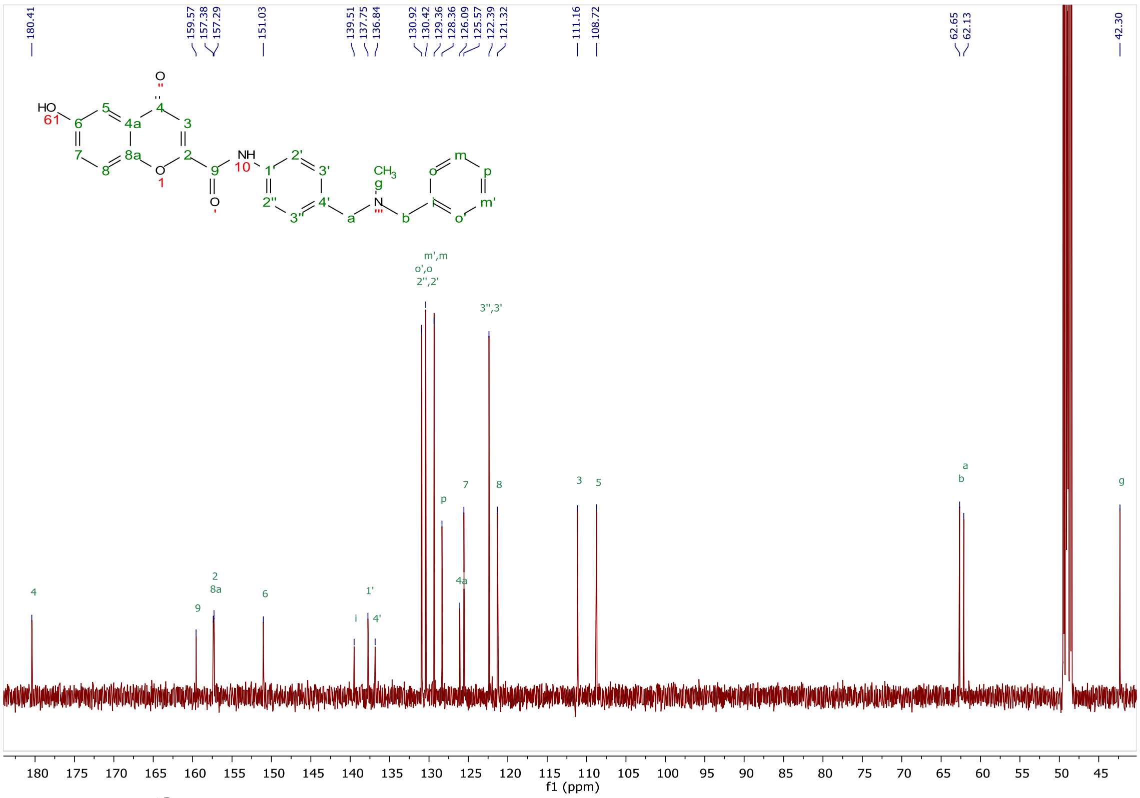

Figure S5. ^{13}C NMR of **2** in MeOD

**Data File**	6347_mev_419_01.d	**Sample Name**	mev_419
**Sample Type**	Sample	**Position**	Vial 7
**Instrument Name**	Instrument 1	**User Name**	
**Acq Method**	ESI_ACN_75_pos.m	**IRM Calibration Status**	Success
**DA Method**	01_busqueda.m	**Comment**	

Compound Table

Compound Label	RT	Mass	Abund	Formula	Tgt Mass	Diff (ppm)
Cpd 1: C25 H22 O4 N2	1.242	414.1583	492443	C25 H22 O4 N2	414.158	0.91

Compound Label	RT	Algorithm	Mass
Cpd 1: C25 H22 O4 N2	1.242	Find By Formula	414.1583

MS Zoomed Spectrum

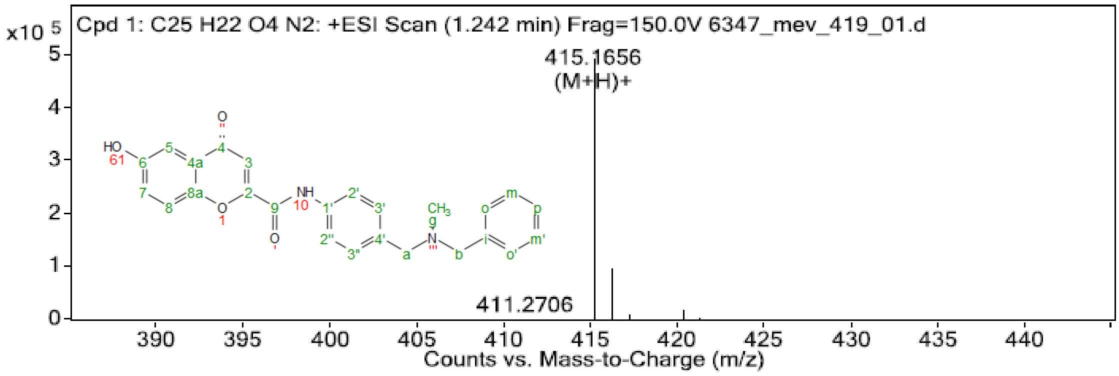

Figure S6. HRMS for compound **2**

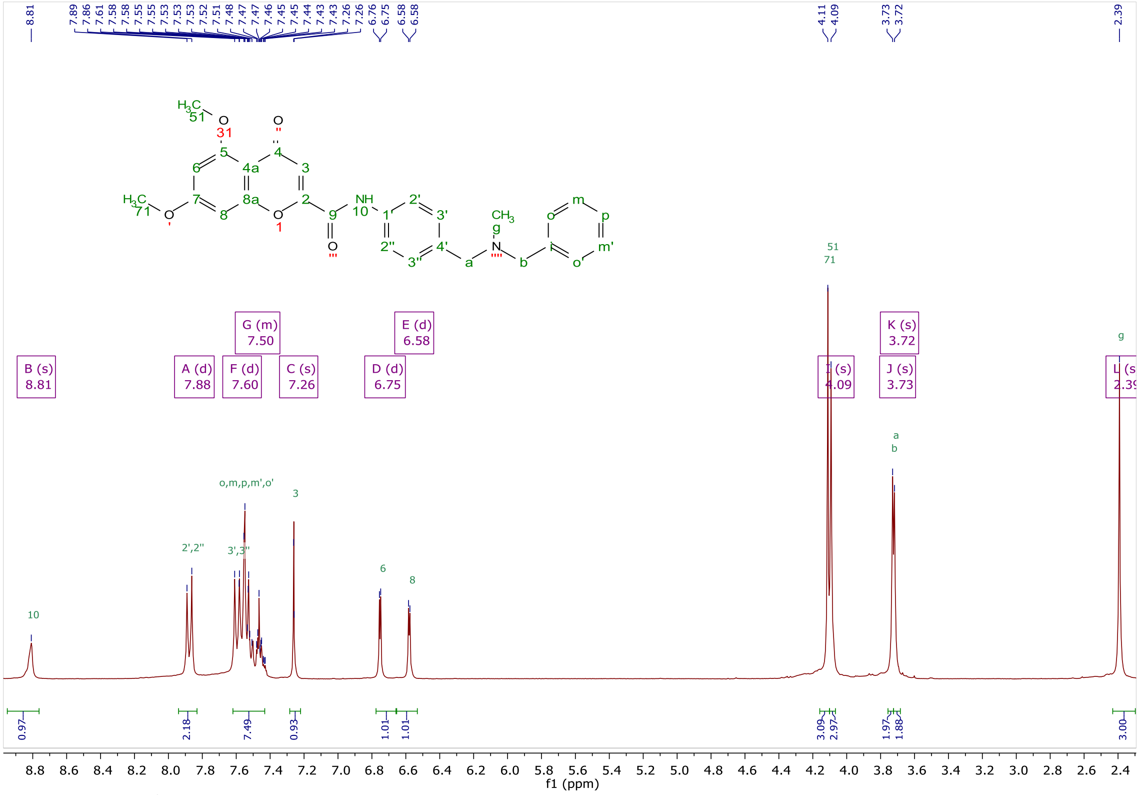

Figure S7. ^{1}H NMR of **3** in $CDCl_3$

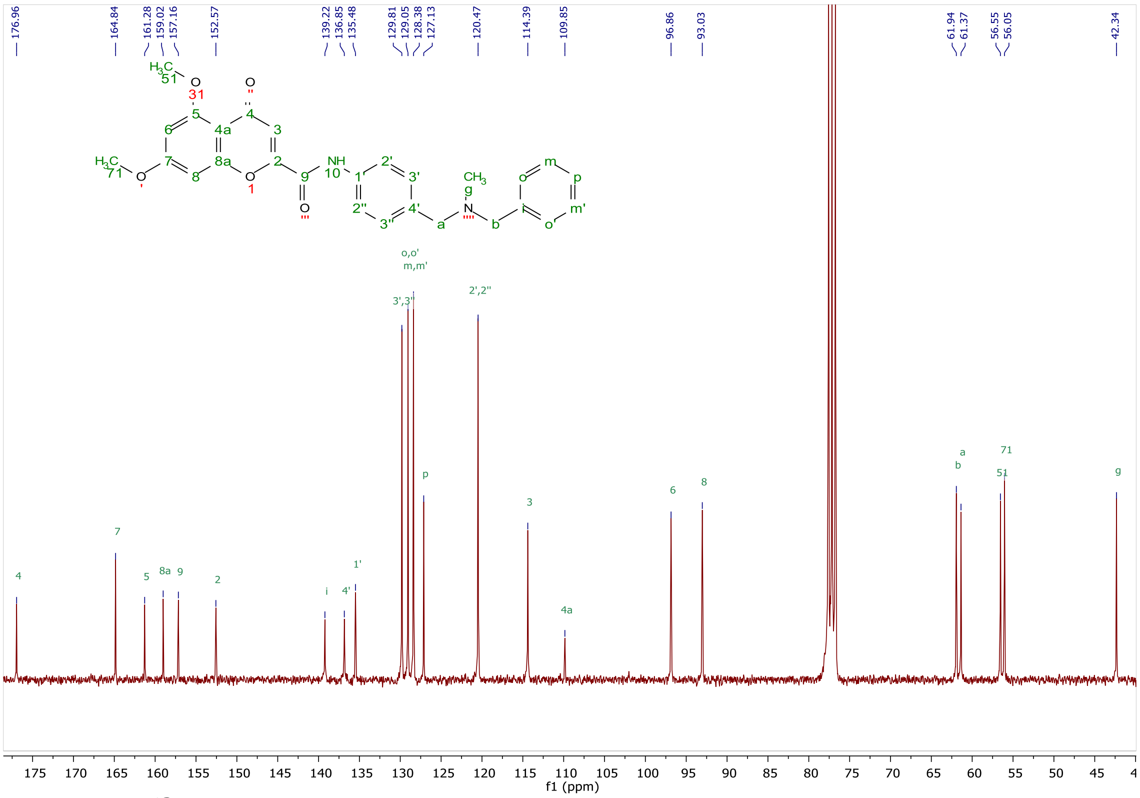

Figure S8. ^{13}C NMR of **3** in $CDCl_3$

**Data File**	5522_mev_304_01.d	**Sample Name**	mev_304
**Sample Type**	Sample	**Position**	Vial 23
**Instrument Name**	Instrument 1	**User Name**	
**Acq Method**	ESI_ACN_75_pos.m	**IRM Calibration Status**	All Ions Missed
**DA Method**	Default.m	**Comment**	

Compound Table

Compound Label	RT	Mass	Abund	Formula	Tgt Mass	Diff (ppm)
Cpd 1: C27 H26 N2 O5	1.15	458.1863	77121	C27 H26 N2 O5	458.1842	4.64

Compound Label	RT	Algorithm	Mass
Cpd 1: C27 H26 N2 O5	1.15	Find By Formula	458.1863

MS Zoomed Spectrum

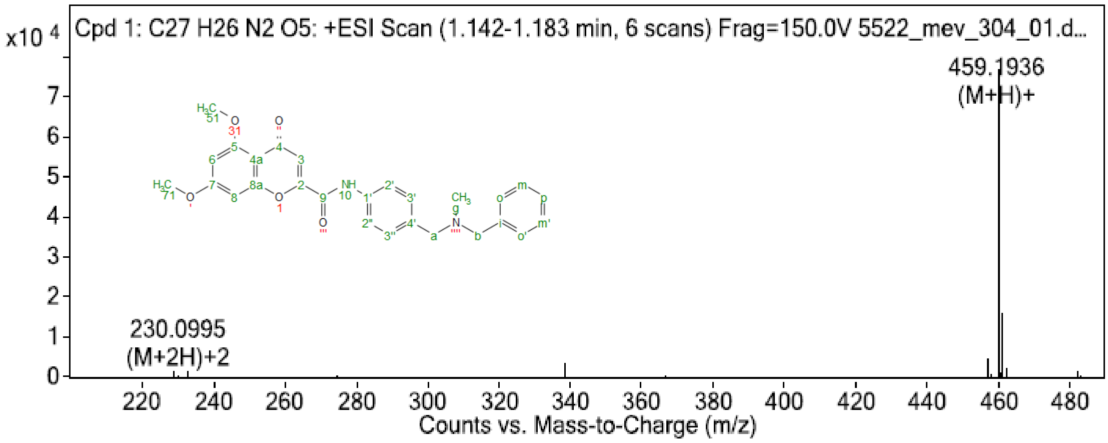

Fig S9. HRMS for compound **3**

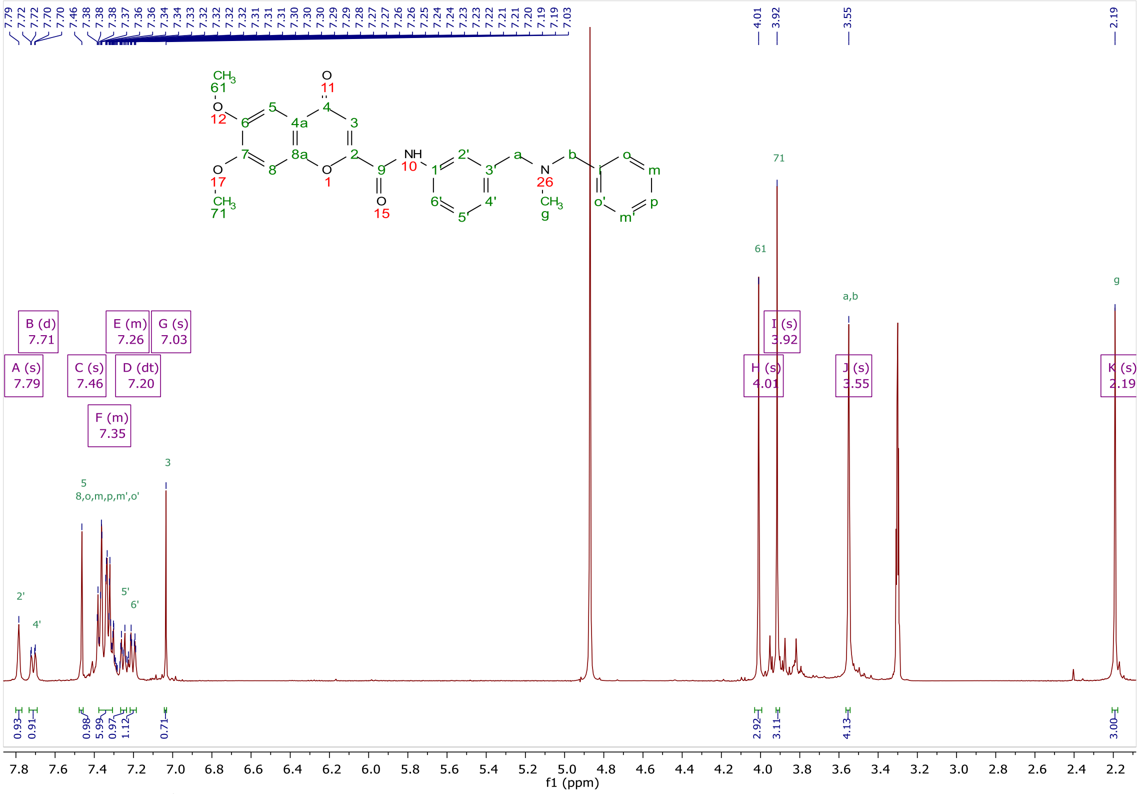

Figure S10. ^{1}H NMR of **6** in MeOD

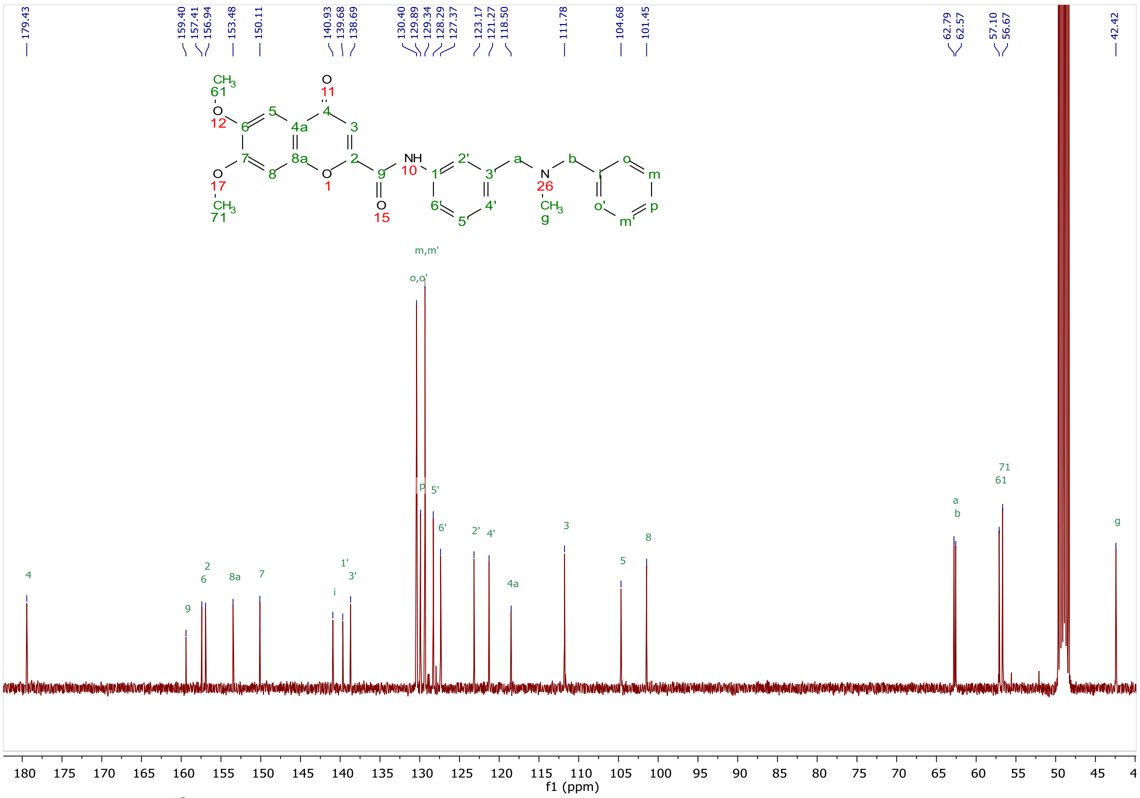

Figure S11. ^{13}C NMR of **6** in MeOD

**Data File**	7789_mev_658_01.d	**Sample Name**	mev_658
**Sample Type**	Sample	**Position**	Vial 3
**Instrument Name**	Instrument 1	**User Name**	
**Acq Method**	ESI_ACN_75_pos.m	**IRM Calibration Status**	Some Ions Missed
**DA Method**	defecto.m	**Comment**	

Compound Table

Compound Label	RT	Mass	Abund	Formula	Tgt Mass	Diff (ppm)
Cpd 1: C27 H26 N2 O5	0.689	458.18405	182379	C27 H26 N2 O5	458.18417	-0.27

Compound Label	RT	Algorithm	Mass
Cpd 1: C27 H26 N2 O5	0.689	Find By Formula	458.18405

MS Zoomed Spectrum

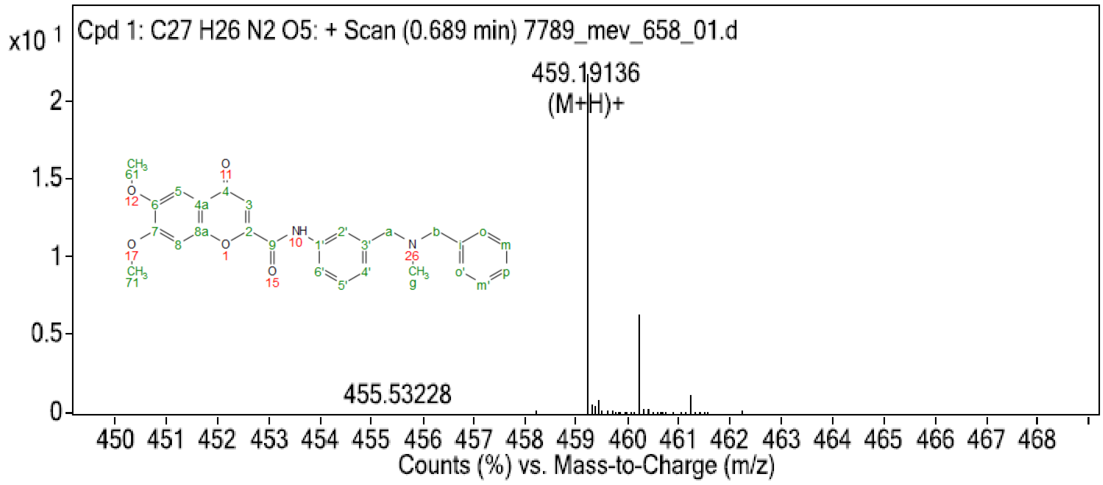

Figure S12. HRMS for compound **6**

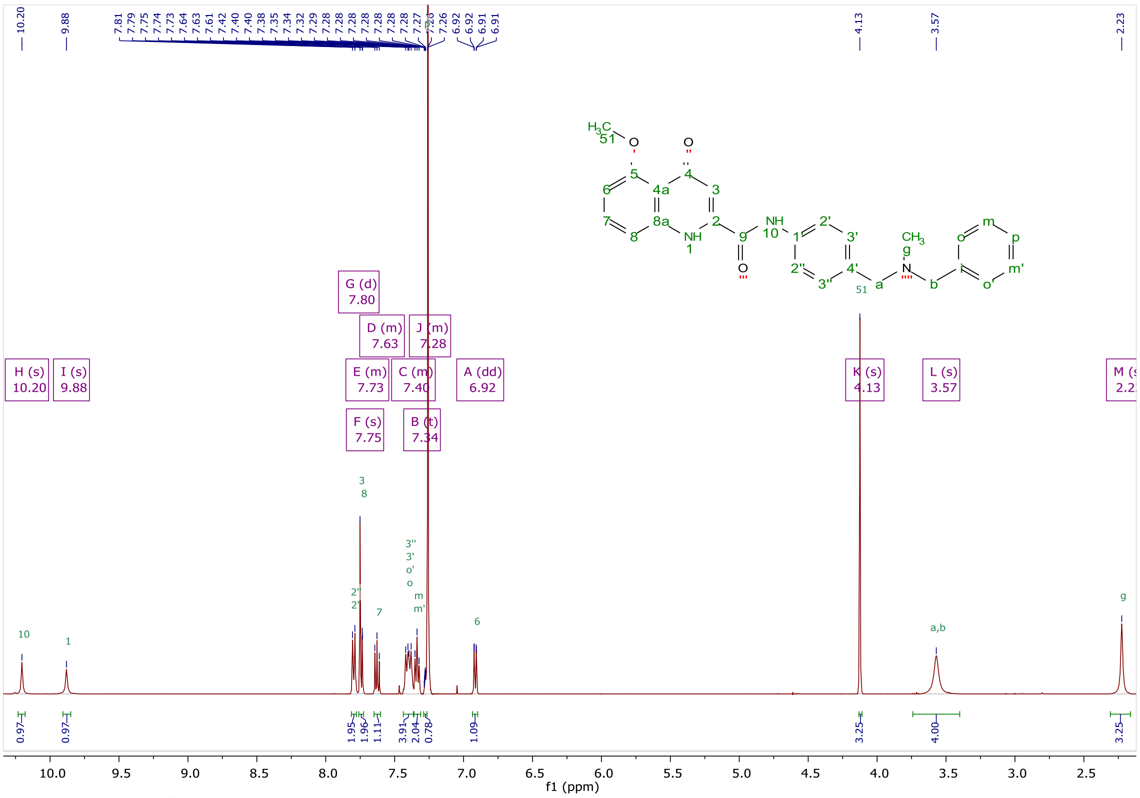

Figure S13. ^{1}H NMR of **7** in $CDCl_3$

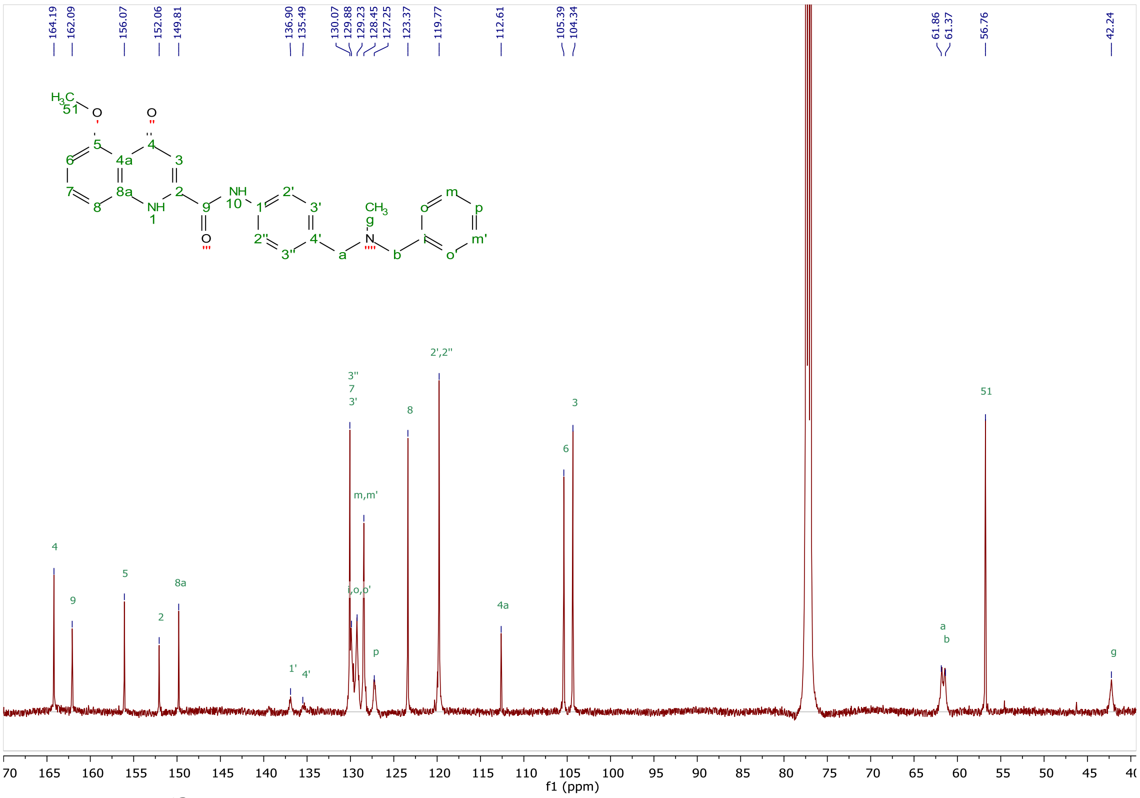

Figure S14. ^{13}C NMR of **7** in $CDCl_3$

**Data File**	7618_mev_454_f1_01.d	**Sample Name**	mev_454_f1
**Sample Type**	Sample	**Position**	Vial 2
**Instrument Name**	Instrument 1	**User Name**	
**Acq Method**	ESI_ACN_75_pos.m	**IRM Calibration Status**	Some Ions Missed
**DA Method**	defecto.m	**Comment**	

Compound Table

Compound Label	RT	Mass	Abund	Formula	Tgt Mass	Diff (ppm)
Cpd 1: C26 H25 N3 O3	0.607	427.19001	618453	C26 H25 N3 O3	427.18959	0.98

Compound Label	RT	Algorithm	Mass
Cpd 1: C26 H25 N3 O3	0.607	Find By Formula	427.19001

MS Zoomed Spectrum

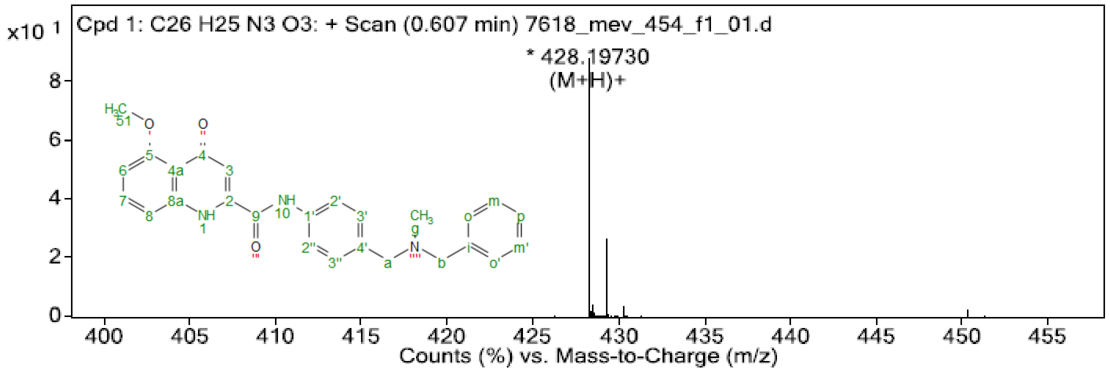

Figure S15. HRMS for Compound **7**

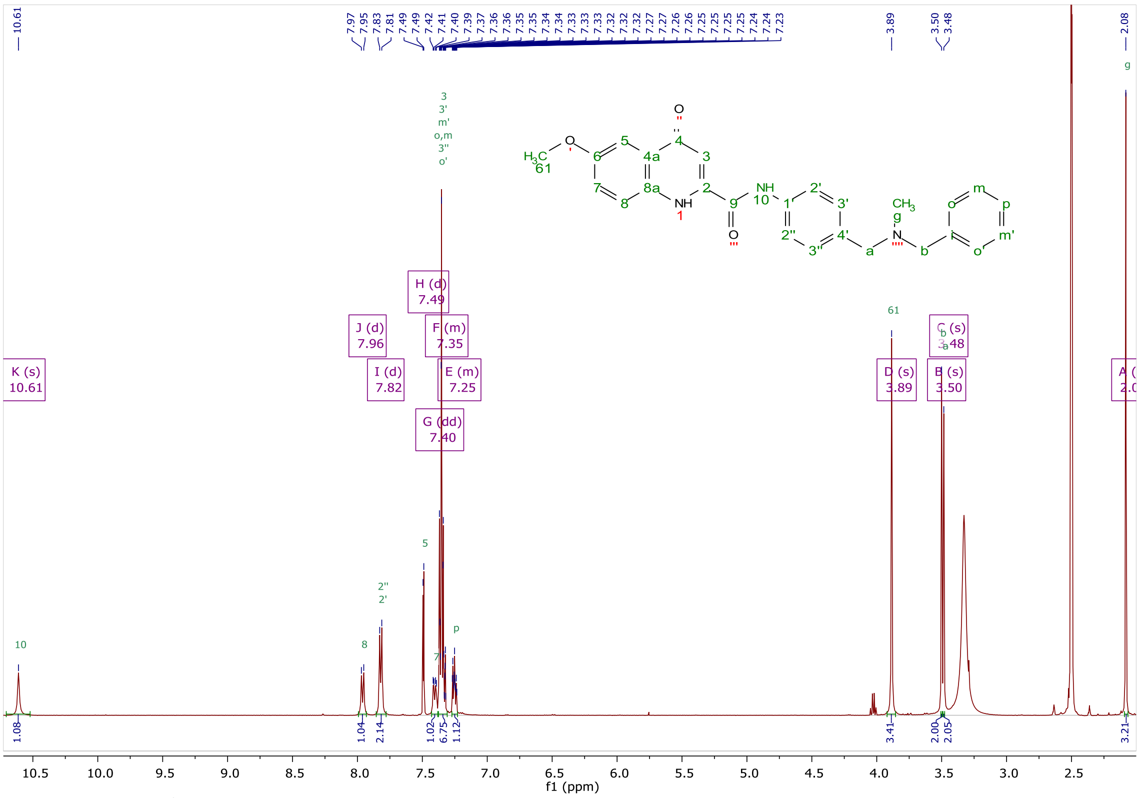

Figure S16. ^{1}H NMR of **8** in DMSO-d6

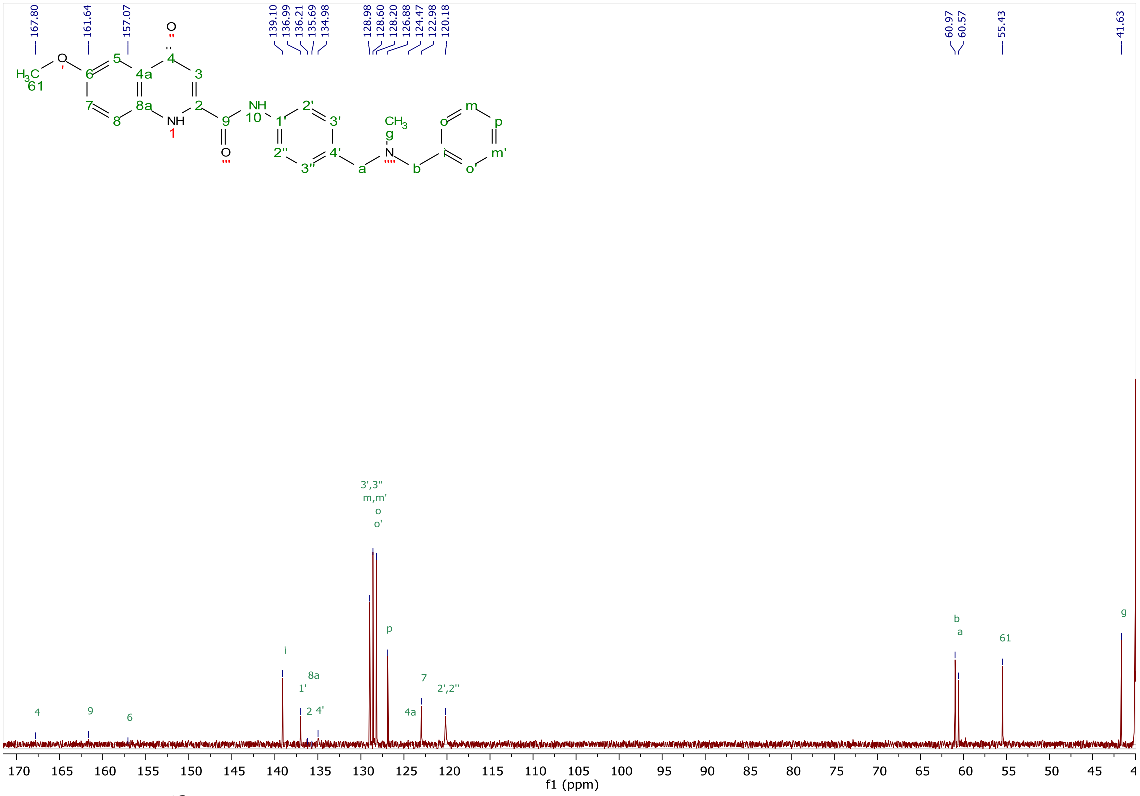

Figure S17. ^{13}C NMR of **8** in DMSO-d_6

**Data File**	6355_mev_456_01.d	**Sample Name**	mev_456
**Sample Type**	Sample	**Position**	Vial 15
**Instrument Name**	Instrument 1	**User Name**	
**Acq Method**	ESI_ACN_75_pos.m	**IRM Calibration Status**	Success
**DA Method**	01_busqueda.m	**Comment**	

Compound Table

Compound Label	RT	Mass	Abund	Formula	Tgt Mass	Diff (ppm)
Cpd 1: C26 H25 O3 N3	0.56	427.1905	344225	C26 H25 O3 N3	427.1896	2.13

Compound Label	RT	Algorithm	Mass
Cpd 1: C26 H25 O3 N3	0.56	Find By Formula	427.1905

MS Zoomed Spectrum

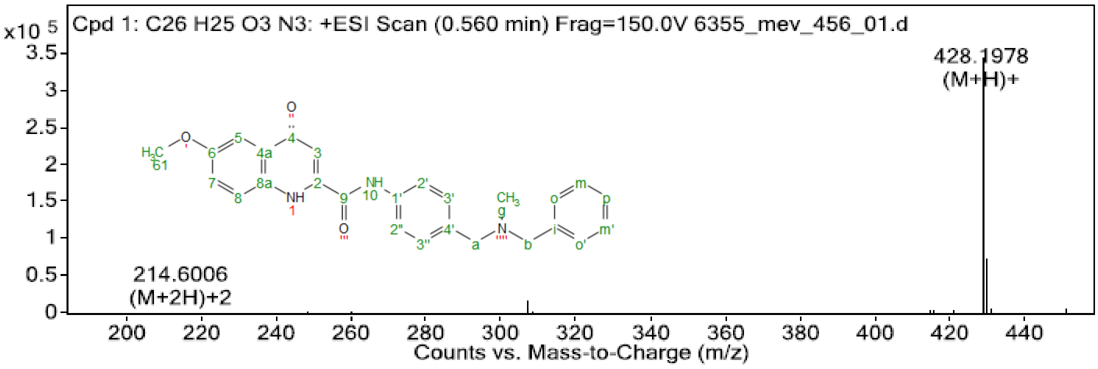

Figure S18. HRMS for compound **8**

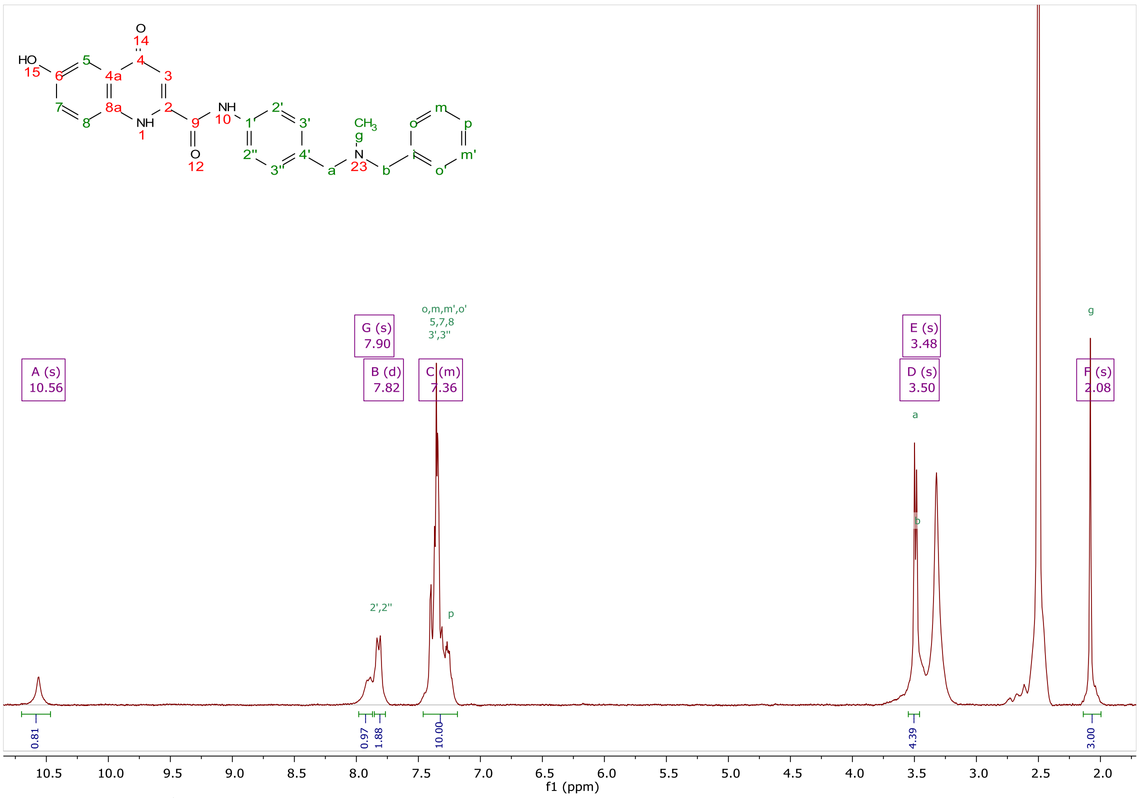

Figure S19. ^{1}H NMR of **9** in DMSO-d_6

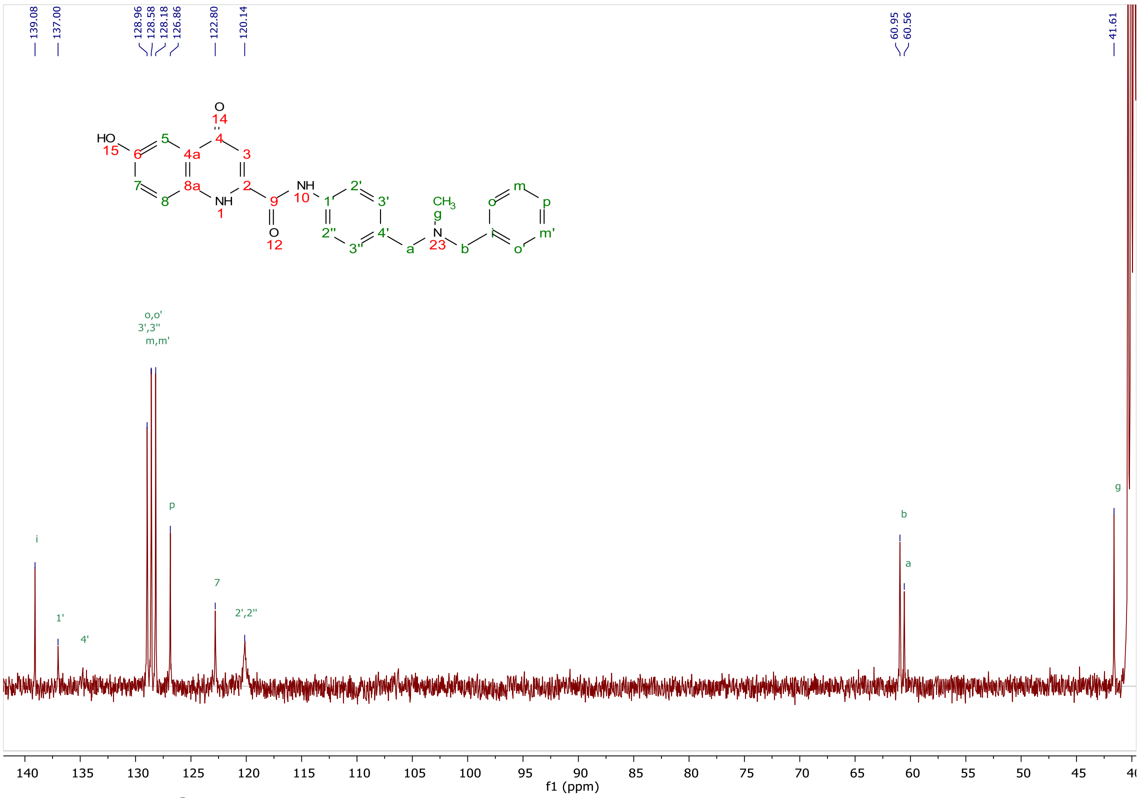

Figure S20. ^{13}C NMR of **9** in DMSO-d6

**Data File**	6354_mev_458_01.d	**Sample Name**	mev_458
**Sample Type**	Sample	**Position**	Vial 14
**Instrument Name**	Instrument 1	**User Name**	
**Acq Method**	ESI_ACN_75_pos.m	**IRM Calibration Status**	Success
**DA Method**	01_busqueda.m	**Comment**	

Compound Table

Compound Label	RT	Mass	Abund	Formula	Tgt Mass	Diff (ppm)
Cpd 1: C25 H23 O3 N3	0.883	413.1751	349781	C25 H23 O3 N3	413.1739	2.84

Compound Label	RT	Algorithm	Mass
Cpd 1: C25 H23 O3 N3	0.883	Find By Formula	413.1751

MS Zoomed Spectrum

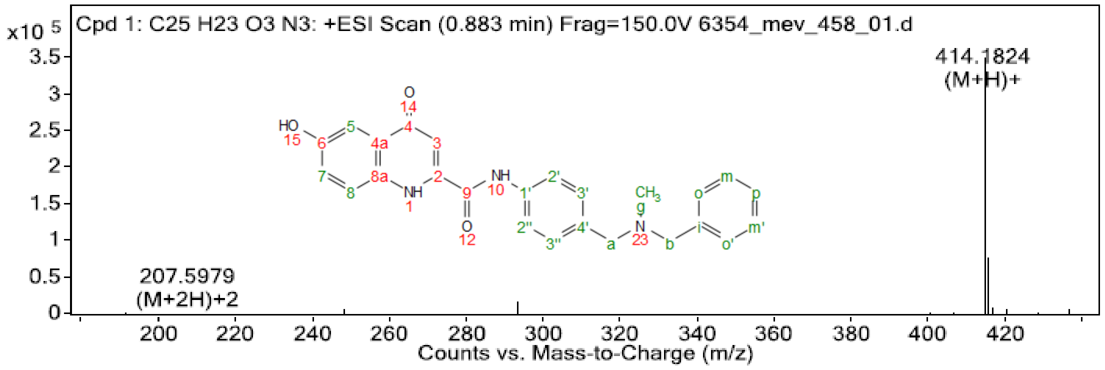

Figure S21. HRMS for compound **9**

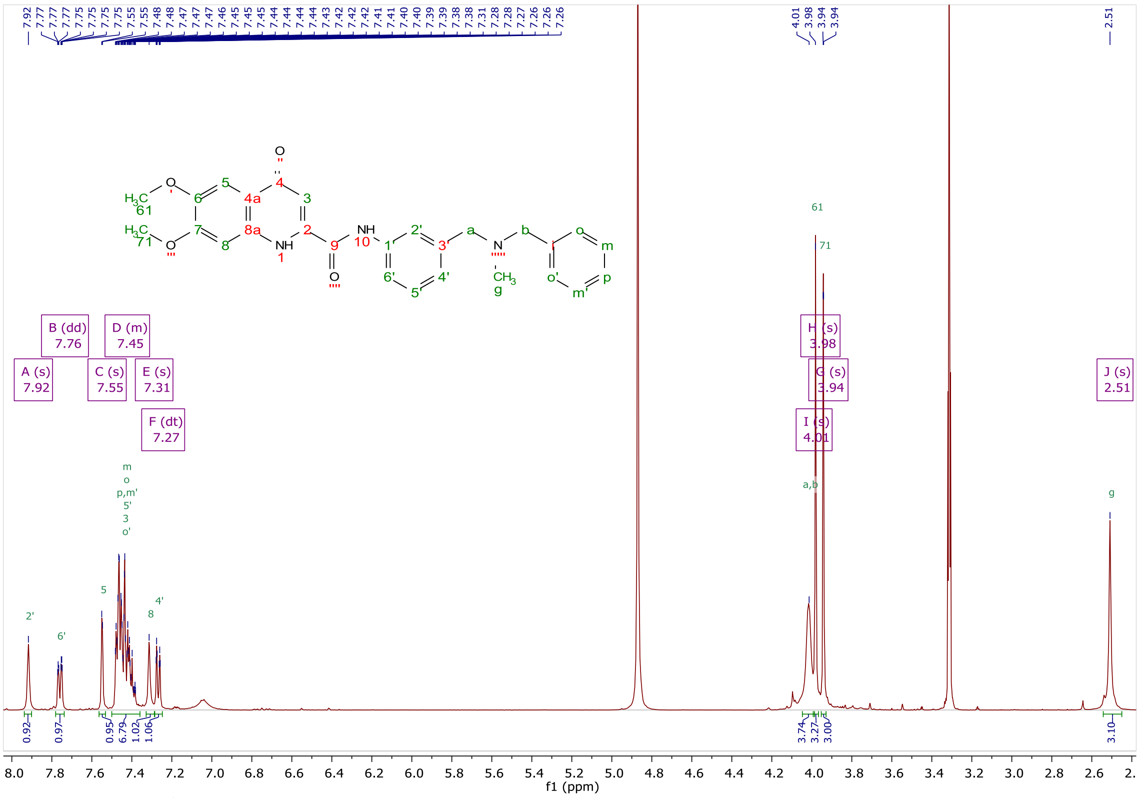

Figure S22. ^{1}H NMR of **13** in MeOD

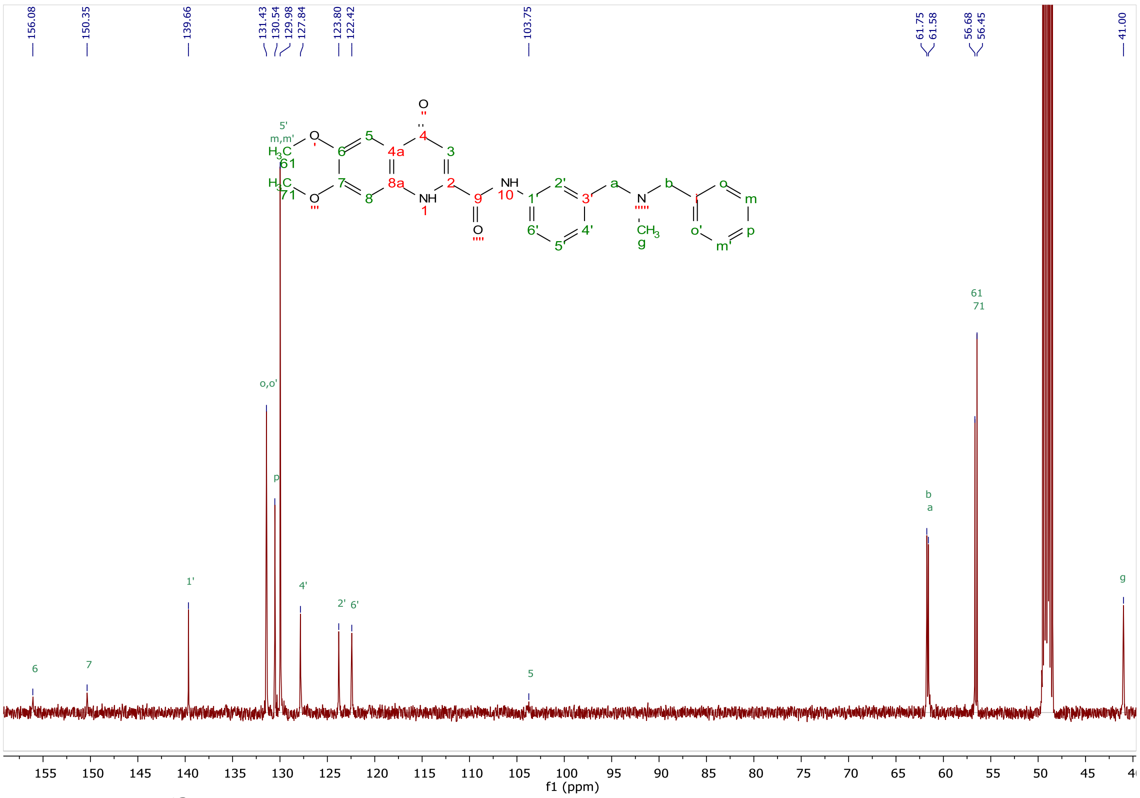

Figure S23. ^{13}C NMR of **13** in MeOD

**Data File**	7788_mev_657_01.d	**Sample Name**	mev_657
**Sample Type**	Sample	**Position**	Vial 2
**Instrument Name**	Instrument 1	**User Name**	
**Acq Method**	ESI_ACN_75_pos.m	**IRM Calibration Status**	Success
**DA Method**	defecto.m	**Comment**	

Compound Table

Compound Label	RT	Mass	Abund	Formula	Tgt Mass	Diff (ppm)
Cpd 1: C27 H27 N3 O4	0.707	457.20024	180025	C27 H27 N3 O4	457.20016	0.17

Compound Label	RT	Algorithm	Mass
Cpd 1: C27 H27 N3 O4	0.707	Find By Formula	457.20024

MS Zoomed Spectrum

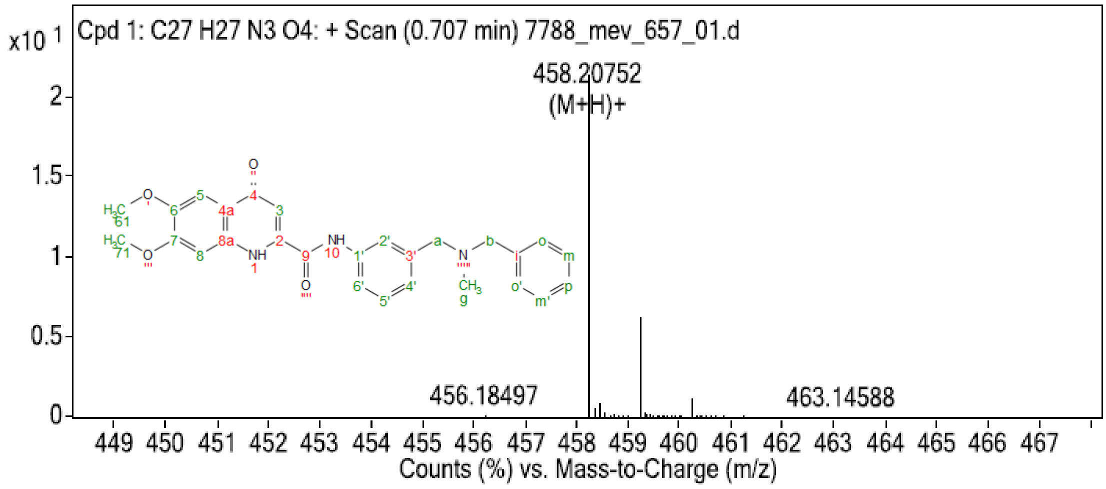

Figure S24. HRMS for compound **13**

References

[1] T. Sterling, J.J. Irwin, ZINC 15--Ligand Discovery for Everyone, J. Chem. Inf. Model. 55 (2015) 2324-2337.

[2] A. Daina, O. Michielin, V. Zoete, SwissADME: a free web tool to evaluate pharmacokinetics, drug-likeness and medicinal chemistry friendliness of small molecules, Sci. Rep. 7 (2017) 42717.

[3] C.A. Marchant, K.A. Briggs, A. Long, In silico tools for sharing data and knowledge on toxicity and metabolism: derek for windows, meteor, and vitic, Toxicol. Mech. Methods 18 (2008) 177-187.
